# Prediction of lateral lymph node metastasis in rectal cancer patients based on MRI using clinical, deep transfer learning, radiomic, and fusion models

**DOI:** 10.3389/fonc.2024.1433190

**Published:** 2024-07-19

**Authors:** Yi Sun, Zhongxiang Lu, Hongjie Yang, Peishi Jiang, Zhichun Zhang, Jiafei Liu, Yuanda Zhou, Peng Li, Qingsheng Zeng, Yu Long, Laiyuan Li, Binbin Du, Xipeng Zhang

**Affiliations:** ^1^ Nankai University, Tianjin, China; ^2^ The Institute of Translational Medicine, Tianjin Union Medical Center of Nankai University, Tianjin, China; ^3^ Department of Colorectal Surgery, Tianjin Union Medical Center, Tianjin, China; ^4^ Tianjin Institute of Coloproctology, Tianjin, China; ^5^ The First Clinical College of Medicine, Gansu University of Traditional Chinese Medicine, Lanzhou, Gansu, China; ^6^ Gansu Provincial Hospital, Gansu Clinical Medical Research Center for Anorectal Diseases, Lanzhou, Gansu, China

**Keywords:** lateral lymph node metastasis, rectal cancer, radiomics, deep transfer learning, predictive model

## Abstract

**Introduction:**

Lateral lymph node (LLN) metastasis in rectal cancer significantly affects patient treatment and prognosis. This study aimed to comprehensively compare the performance of various predictive models in predicting LLN metastasis.

**Methods:**

In this retrospective study, data from 152 rectal cancer patients who underwent lateral lymph node (LLN) dissection were collected. The cohort was divided into a training set (n=86) from Tianjin Union Medical Center (TUMC), and two testing cohorts: testing cohort (TUMC) (n=37) and testing cohort from Gansu Provincial Hospital (GSPH) (n=29). A clinical model was established using clinical data; deep transfer learning models and radiomics models were developed using MRI images of the primary tumor (PT) and largest short-axis LLN (LLLN), visible LLN (VLLN) areas, along with a fusion model that integrates features from both deep transfer learning and radiomics. The diagnostic value of these models for LLN metastasis was analyzed based on postoperative LLN pathology.

**Results:**

Models based on LLLN image information generally outperformed those based on PT image information. Rradiomics models based on LLLN demonstrated improved robustness on external testing cohorts compared to those based on VLLN. Specifically, the radiomics model based on LLLN imaging achieved an AUC of 0.741 in the testing cohort (TUMC) and 0.713 in the testing cohort (GSPH) with the extra trees algorithm.

**Conclusion:**

Data from LLLN is a more reliable basis for predicting LLN metastasis in rectal cancer patients with suspicious LLN metastasis than data from PT. Among models performing adequately on the internal test set, all showed declines on the external test set, with LLLN_Rad_Models being less affected by scanning parameters and data sources.

## 1 Introduction

Lateral lymph node (LLN) metastasis is a significant route of metastasis for mid- and low rectal cancers, a 20.1% rate of metastases (1). Current treatment strategies for suspected LLN metastasis include: 1. total mesorectal excision (TME) after neoadjuvant chemoradiotherapy (nCRT); 2. TME combined with lateral lymph node dissection (LLND); and 3. TME combined with LLND after nCRT (2, 3). Accurate diagnosis of LLN metastasis is crucial for determining the appropriate treatment strategy. Preoperative pathological or cytological evidence of LLNs is difficult to obtain; hence, the diagnosis of LLN metastasis primarily relies on imaging studies. The short-axis diameter of the lymph node is the most critical parameter for assessing the presence of metastasis (4). Immune responses induced by tumors can also lead to lymph node enlargement, which does not necessarily indicate tumor cell metastasis. In contrast, nonmetastatic lymph node enlargement is an indicator of better long-term prognosis in colorectal cancer (CRC) patients (5). Currently, commonly used imaging methods for diagnosing lymph node metastasis include MRI, CT, positron emission tomography (PET)/CT, and endorectal ultrasound. These imaging techniques demonstrate relatively low sensitivity and specificity in determining the nature of lymph nodes (6–8).

Over the past decade, the field of radiomics has established itself as an important technique in quantitative image analysis. Radiomics involves the extraction of a large number of quantitative features from medical images using sophisticated data characterisation algorithms (9). These features can then be used to build predictive models of clinical outcomes, improving the accuracy of medical diagnoses and treatment plans (10). While radiomics focuses on pre-defined features extracted from images, deep learning approaches, particularly deep transfer learning models, have gained popularity for their ability to automatically learn features from data. Deep transfer learning uses pre-trained neural networks that can be fine-tuned to specific medical imaging tasks, reducing the need for large labelled datasets (11). Radiomics and deep transfer learning technologies have demonstrated exceptional capabilities in disease diagnosis, molecular typing, and predicting treatment responses (12). Studies have shown that in the diagnosis of rectal cancer lymph node metastasis, radiomics models exhibit greater diagnostic efficacy than traditional imaging methods (13). This approach aimed to explore optimal methods for constructing machine learning diagnostic models for detecting LLN metastasis in rectal cancer patients suspected LLN metastasis.

## 2 Methods

### 2.1 Study cohort

In this study, data from 152 rectal cancer patients whose MRI-documented LLNs exceeded 5 mm in short-axis diameter were retrospectively collected, all of whom had undergone LLND. A clinical diagnostic model was constructed, along with seven other models developed specifically for LLNs and primary tumor (PT). Three types of models were developed for both largest short-axis LLN (LLLN)and PT: a deep transfer learning (DTL) diagnostic model, a radiomic model, and a fusion model that integrates features from both DTL and radiomics. Additionally, a radiomic model was developed based on visible LLN (VLLN). Written informed consent was waived in this retrospective study. The study protocol was approved by the Tianjin Union Medical Center (TUMC)’s Ethics Committee (Approval No. 2022-C23) and Gansu Provincial Hospital (GSPH)’s Ethics Committee (Approval No. 2024-243). Clinical and imaging data of rectal cancer patients who met the following criteria were collected from June 2017 to May 2024. The inclusion criteria were as follows: 1. Patients who underwent LLND surgery at the same time as TME surgery and who had pathologically confirmed rectal cancer; 2. Patients with pelvic MR images and LLNs with short-axis diameters exceeding 5 mm on MRI, as assessed by the surgical team preoperatively; The exclusion criteria were as follows: 1. Patients without T2WI data. 2. Patients without complete clinical and pathological information.3. Patients for which the LLNs were not visible in horizontal T2WI images because they were outside the field of view of the scan, even though LLNs greater than 5 mm in the short axis could be detected in sagittal or coronal positions. 4. In those who received nCRT, induction neoadjuvant chemotherapy or consolidation neoadjuvant chemotherapy before surgery, those with pathologically negative LN were excluded to account for potential curative treatment of nCRT and the subsequent effect on modeling.

According to the postoperative pathological results of the LLNs in the patients, the patients were divided into two groups: the LLN metastasis group, consisting of patients with one or more pathologically positive LLNs, and the non-LLN metastasis group, consisting of patients with zero pathologically positive LLNs. Patients from TUMC were randomly divided at a 7:3 ratio into a training cohort (TUMC) (n=86) and a testing cohort (TUMC) (n=37). Patients from GSPH were designated as testing cohort (GSPH) (n=29).

### 2.2 Region of interest segmentation

We obtained MR-T2W images of the pelvis at admission from the image archiving and communication system at Tianjin Union Medical Center. The horizontal MR-T2W images obtained from the patient cohort were exported to the 3D Slicer program (v.5.2.2) for ROI segmentation. A radiologist with more than five years of experience in the field utilized this software to accurately delineate the boundaries of the PT and the VLLN.

### 2.3 Radiomics feature extraction

In our study, we utilized PyRadiomics to extract a total of 1,198 radiomics features from the PT and the each VLLN. The extracted features include first-order features, shape-based features, and various texture features categorized into a gray-level co-occurrence matrix (GLCM), gray-level dependence matrix (GLDM), gray-level run length matrix (GLRLM), gray-level size zone matrix (GLSZM), and neighborhood gray-tone difference matrix (NGTDM). The proportions of each category are illustrated in **Supplementary Figure S1**. The detailed parameters used for radiomic feature extraction are described in the **Supplementary Materials** and can also be found on the PyRadiomics website (https://pyradiomics.readthedocs.io/en/latest/). The configuration file for feature extraction is provided in the **Supplementary File**. Radiomics features from PT were used to construct PT_Rad_Models (radiomics models based on primary tumor). Radiomics features from the LLLN were used to construct LLLN_Rad_Models (radiomics models based on largest short-axis lateral lymph node). The maximum, minimum, mean, median value (when the number of VLLN is even, the median value is equal to the mean), and standard deviation of each feature of all VLLN of each participant were recorded, resulting in a total of 5990 radiomics features obtained from each patient for VLLN_Rad_Models (radiomics models based on all visible lateral lymph nodes).

### 2.4 Radiomics feature selection and model construction

The radiomics features were standardized using z score normalization. We also conducted Mann−Whitney U tests and feature screening for all radiomic features. Only radiomic features with p values < 0.05 were retained. To handle strong correlations between features (Spearman correlation coefficient ≥ 0.9), we employed a greedy recursive feature deletion strategy for feature filtering. This strategy entails iteratively removing the feature with the highest redundancy within the current feature set until the current set no longer contains features with a correlation coefficient greater than 0.9. To further refine the features, multivariate least absolute shrinkage and selection operator (LASSO) regression was employed. After LASSO feature selection, we conducted supervised learning using eight diverse machine learning classifiers, including random forest (RF), k-nearest neighbor (KNN), logistic regression (LR), multilayer perceptron (MLP), support vector machine (SVM), extreme gradient boosting (XGBoost), light gradient boosting machine (LightGBM), and ExtraTrees. Twenty-four models were constructed, with eight PT_Rad_Models and eight LLLN_Rad_Models, and eight VLLN_Rad_Models.

### 2.5 Clinical model construction

The clinical characteristics and radiological features in **Table 1** were used to construct the clinical model. These features were standardized using z score normalization. Next, feature selection was performed using t tests and chi-square tests (P < 0.10) to screen for clinical risk factors for LLN metastasis in the training set, followed by training eight diverse machine learning classifiers.

**Table 1:** Characteristics of patients in the training and test cohorts.

Characteristic	Training cohort (TUMC, n=86)			Test cohort (TUMC, n=37)			Test cohort (GSPH, n=29)		
	Negative for LLN metastasis	Positive for LLN metastasis	P value	Negative for LLN metastasis	Positive for LLN metastasis	P value	Negative for LLN metastasis	Positive for LLN metastasis	P value
Age, mean ± SD, years	60.16 ± 13.30	59.55 ± 12.32	0.68	62.06 ± 8.04	56.43 ± 12.01	0.11	61.90 ± 7.36	54.74 ± 9.88	0.05
Height, mean ± SD, cm	168.65 ± 8.11	166.55 ± 8.26	0.24	167.75 ± 6.86	166.05 ± 7.93	0.50	165.92 ± 5.98	166.85 ± 7.17	0.73
Weight, mean ± SD, kg	69.73 ± 13.73	67.23 ± 12.37	0.38	68.25 ± 12.42	67.12 ± 11.68	0.78	63.00 ± 11.28	65.16 ± 9.32	0.59
CEA, mean ± SD, ng/mL	14.47 ± 25.52	15.01 ± 17.49	0.15	203.55 ± 506.53	19.61 ± 29.61	0.68	9.95 ± 15.42	16.81 ± 48.06	0.57
CA19-9, mean ± SD, ng/mL	91.12 ± 270.73	61.61 ± 156.86	0.54	32.31 ± 46.45	23.74 ± 22.42	0.88	8.92 ± 8.81	122.82 ± 296.80	0.02
Distance to anal margin, mean ± SD, cm	4.71 ± 2.65	3.94 ± 1.86	0.09	4.67 ± 1.81	4.67 ± 2.38	1.00	5.38 ± 2.58	4.21 ± 1.84	0.17
Lesion length, mean ± SD, cm	4.25 ± 1.49	4.77 ± 2.37	0.20	4.77 ± 1.72	4.50 ± 1.40	0.60	4.83 ± 2.89	4.70 ± 2.34	0.90
NoEMLN, mean ± SD	1.59 ± 1.52	1.96 ± 1.97	0.58	1.62 ± 1.59	1.67 ± 1.59	0.92	3.20 ± 2.57	3.47 ± 2.39	0.94
NoELLN, mean ± SD	1.41 ± 0.86	1.86 ± 1.12	0.01	1.12 ± 0.34	1.67 ± 1.11	0.12	1.40 ± 0.70	1.68 ± 1.06	0.67
Gender, n (%)			0.47			0.50			1.00
female	10(27.03)	18(36.73)		5(31.25)	10(47.62)		4(40.00)	9(47.37)	
male	27(72.97)	31(63.27)		11(68.75)	11(52.38)		6(60.00)	10(52.63)	
T stage, n (%)			0.66			0.85			0.40
2	2(5.41)	5(10.20)		1(6.25)	2(9.52)		1(10.00)	1(5.26)	
3	29(78.38)	38(77.55)		12(75.00)	14(66.67)		8(80.00)	14(73.68)	
4	6(16.22)	6(12.24)		3(18.75)	5(23.81)		1(10.00)	4(21.05)	
N stage, n (%)			0.53			0.29			0.74
1	23(62.16)	26(53.06)		12(75.00)	11(52.38)		4(40.00)	5(26.32)	
2	14(37.84)	23(46.94)		4(25.00)	10(47.62)		6(60.00)	14(73.68)	
CRM, n (%)			0.26			1.00			1.00
0	19(51.35)	18(36.73)		4(25.00)	5(23.81)		4(40.00)	7(36.84)	
1	18(48.65)	31(63.27)		12(75.00)	16(76.19)		6(60.00)	12(63.16)	
EMVI, n (%)			0.35			0.63			0.80
0	22(59.46)	23(46.94)		9(56.25)	9(42.86)		6(60.00)	9(47.37)	
1	15(40.54)	26(53.06)		7(43.75)	12(57.14)		4(40.00)	10(52.63)	

LLN, lateral lymph node; CEA, carcinoembryonic antigen; CA19-9, carbohydrate antigen 19-9; CRM, circumferential resection margin; EMVI, extramural vascular invasion; NoELLN, number of enlarged lateral lymph nodes; NoEMLN, number of enlarged mesorectal lymph nodes; enlarged lymph nodes indicate a short-axis diameter of lateral lymph nodes ≥5 mm; TUMC, Tianjin Union Medical Center; GSPH, Gansu Provincial Hospital.

### 2.6 DTL model development and feature extraction

For PT and LLLN, the layer with the largest ROI area was selected. In that layer, the ROI area with the smallest bounding rectangle was saved as a PNG image. The ResNet18 network was pretrained using the ImageNet dataset, and transfer learning was subsequently performed on the training set. ImageNet is a large-scale image database that contains millions of labeled images across thousands of categories. ImageNet-based transfer learning has been used in many medical studies. We employed a global fine-tuning strategy to update the parameters, thereby adapting ResNet18 for the prediction of LLN metastasis. The learning rate was set to 0.005, the number of epochs was set to 50, and the Adam optimizer was used to update the parameters. Two models were constructed: PT_DTL_ResNet18 (deep transfer learning on primary tumor using ResNet18) and LLLN_DTL_ResNet18 (deep transfer learning on largest short-axis lateral lymph node using ResNet18). The trained ResNet18 could be used to predict the probability of LLN metastasis for each rectangular image.

After completing the training of ResNet18, we utilized ResNet18 to extract 512 deep learning features of each patch from the penultimate average pooling layer in ResNet18.

### 2.7 Construction of the fusion model

This study employed feature-level fusion strategies to establish a fusion model. Feature-level fusion, also known as early fusion, involves connecting all features from different modalities into a single feature vector. The radiomics features of the primary tumor were extracted using PyRadiomics, while the deep learning (DL) features were obtained through ResNet18, as described above. These DL and radiomics features were standardized using z score normalization. Subsequently, U tests, Spearman correlation analyses, and LASSO analyses were performed to select the features, followed by training eight diverse machine learning classifiers. Sixteen models were constructed, with eight PT_Fusion_Models (the models combine radiomics and deep transfer learning features based on the primary tumor) and eight LLLN_Fusion_Models (the models combine radiomics and deep transfer learning features based on the largest short-axis lateral lymph node).

### 2.8 Model validation and comparison

**Figure 1:**
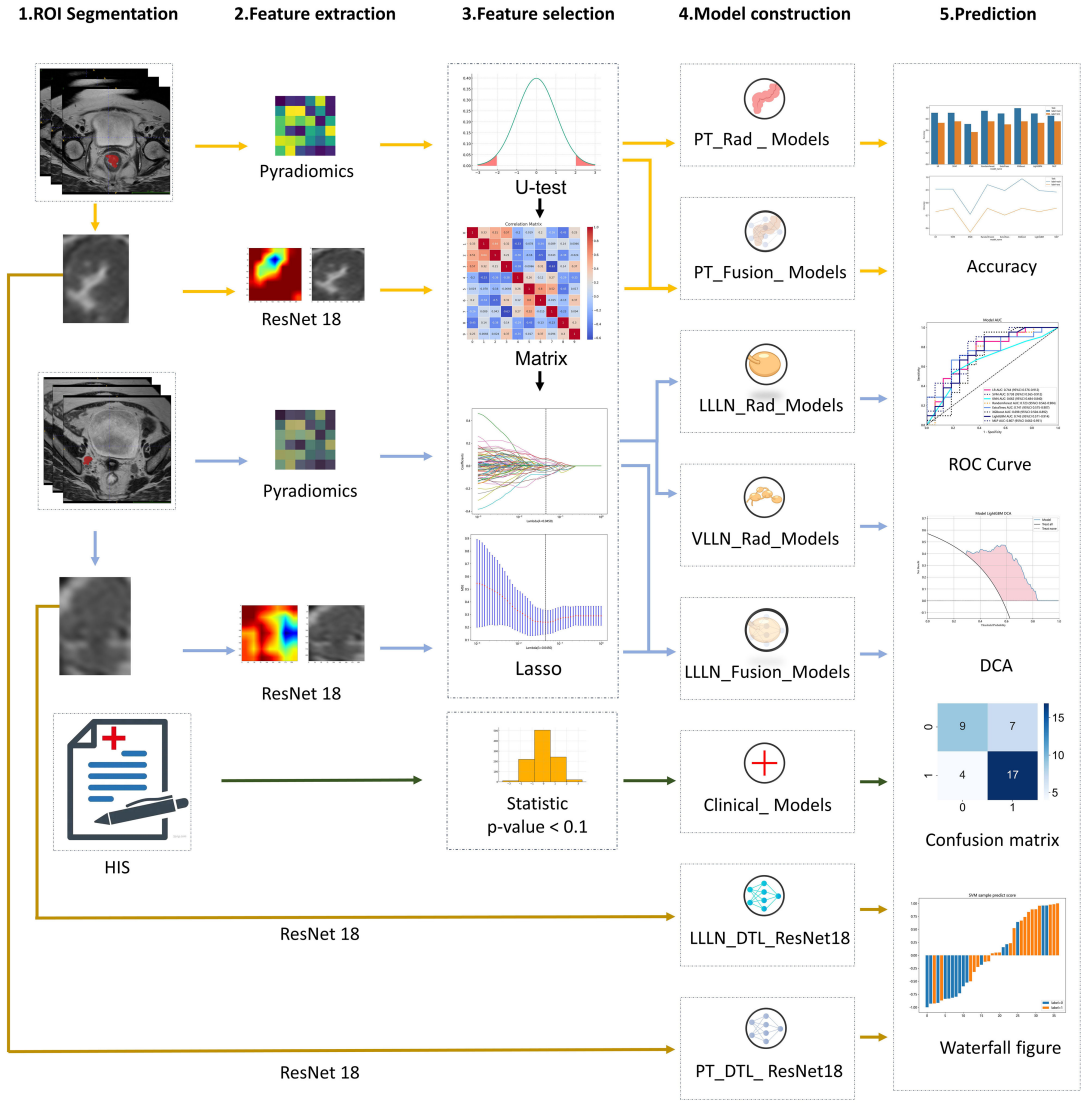
The workflow of the clinical, radiomic, DTL (ResNet18), and fusion (radiomics and DTL) models. (DTL, deep transfer learning; PT, primary tumor; LLLN, largest short-axis lateral lymph node; VLLN, visible lateral lymph nodes; PT_Rad_Models, radiomics model based on primary tumor; PT_Fusion_Models, the models combine radiomics and deep transfer learning features based on the primary tumor; LLLN_Rad_Models, the radiomics model based on largest short-axis lateral lymph node; VLLN_Rad_Models, the radiomics model based on all visible lateral lymph nodes; LLLN_Fusion_Models, the models combine radiomics and deep transfer learning features based on largest short-axis lateral lymph node; PT_DTL_ResNet18, deep transfer learning on primary tumor using ResNet18; LLLN_DTL_ResNet18, deep transfer learning on largest short-axis lateral lymph node using ResNet18; ROC, receiver operating characteristic; DCA, decision curve analysis; HIS, Hospital Information System).

After construction, the prediction model was validated in the testing cohort (TUMC) and the testing cohort (GSPH). The sensitivity, specificity, precision, and F1 score were measured to evaluate the diagnostic accuracy. Additionally, a confusion matrix and a waterfall figure were used for further comparison. Receiver operating characteristic (ROC) curves and the area under the curve (AUC) were generated to evaluate the discrimination performance of the prediction model. Decision curve analysis (DCA) was performed to assess the clinical utility and net benefit of the model. The flowchart of the study is illustrated in **Figure 1**.

## 3 Results

### 3.1 Baseline characteristics and clinical model analysis

This study involved a cohort of 152 patients with a mean age of 59.09 years ( ± 11.6 years). The sex distribution revealed that 63.8% of patients were male and 36.2% were female. Among the 123 patients from TUMC, 57% (70/123) had pathological LLN positivity, and of these 70 patients, 24% (17/70) underwent nCRT treatment. From the GSPH cohort of 29 patients, 66% (19/29) had pathological LLN positivity, and of these 19 patients, 58% (11/19) underwent nCRT treatment. (Detailed clinical information of the patient can be found in **Supplementary Materials 2**). The baseline clinical characteristics are presented in **Table 1**. According to the t test and chi-square test, two characteristics had p values less than 0.1 in the training cohort (TUMC): distance to the anal margin (p = 0.09) and the number of enlarged mesorectal lymph nodes (NoELLNs) (p = 0.01). The clinical models will be constructed based on these two characteristics.

### 3.2 Feature selection

#### 3.2.1 Primary tumor radiomic features

**Figure 2:**
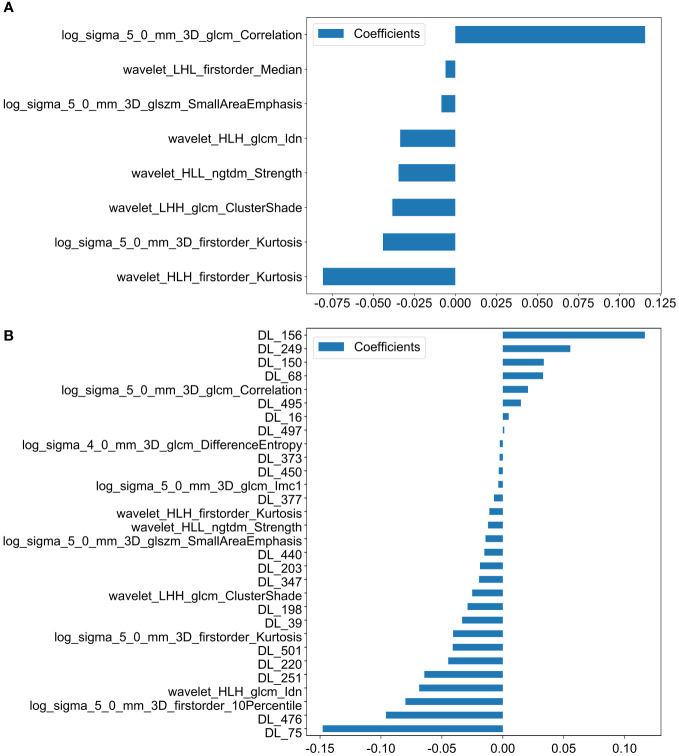
Histogram of scores based on the selected features after LASSO regression. **(A)** Features based on PT radiomics. **(B)** Features based on radiomics and DTL (ResNet18) from PT. (PT, primary tumor; DTL, deep transfer learning; glcm, gray−level co−occurrence matrix; gldm, gray-level dependence matrix; glrlm, gray−level run length matrix; glszm, gray−level size zone matrix; ngtdm, neighborhood gray−tone difference matrix; Imc2, informational measure of correlation 2; DL, deep learning; HLH, high-low-high-pass filtered image; LHH, low-high-high-pass filtered image; HLL, high-low-low-pass filtered image).

We ultimately identified 8 key radiomic features of the primary tumor (PT) of the 1,198 radiomic features (**Figure 2A**). These features were selected specifically for constructing the PT_Rad_Models.

#### 3.2.2 Primary tumor fusion features

30 Fusion features for the primary tumor, which included 10 key radiomic features out of 1,198 radiomic features and 20 key deep learning features out of 512 deep learning features (**Figure 2B**). These features were utilized to develop the PT_Fusion_Models.

#### 3.2.3 LLLN radiomic features

**Figure 3:**
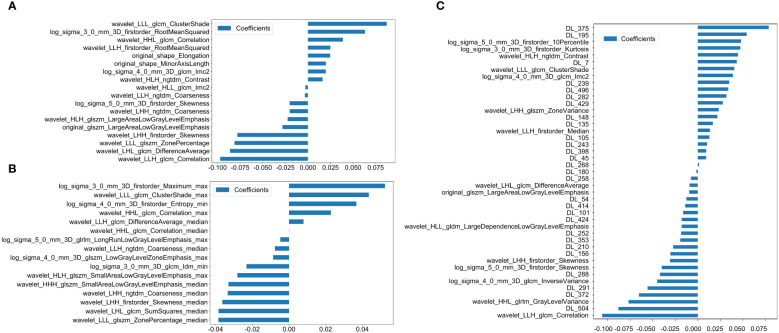
Histogram of the scores based on the selected features after LASSO regression. **(A)** Features based on radiomics from LLLN. **(B)** Features based on radiomics from VLLN **(C)** Features based on radiomics and DTL (ResNet18) from LLLN. (LLLN, largest short-axis lateral lymph node; VLLN, visible lateral lymph nodes; PT, primary tumor; DTL, deep transfer learning; glcm, gray−level co−occurrence matrix; gldm, gray-level dependence matrix; glrlm, gray−level run length matrix; glszm, gray−level size zone matrix; ngtdm, neighborhood gray−tone difference matrix, Imc2, informational measure of correlation 2; DL, deep learning; LLH, low-low-high-pass filtered image; LHL, low-high-low-pass filtered image; LLL, low-low-low-pass filtered image; LHH, low-high-high-pass filtered image; HLH, high-low-high-pass filtered image; HLL, high-low-low-pass filtered image; HHL, high-high- low-pass filtered image; HHL, high-high-low-pass filtered image).

We ultimately identified 18 key radiomic features of the LLLN out of 1,198 radiomic features (**Figure 3A**). These features were selected specifically for constructing the LLLN_Rad_Models.

#### 3.2.4 VLLN radiomic features

We ultimately identified 16 key radiomic features of the VLLN out of 5990 radiomic features (**Figure 3B**). These features were selected specifically for constructing the LLLN_Rad_Models.

#### 3.2.5 LLLN fusion features

43 fusion features for the LLN, which included 15 key radiomic features out of 1,198 radiomic features and 28 key deep learning features out of 512 deep learning features (**Figure 3C**). These features were utilized to develop the LLLN_Fusion_Models.

The complete set of feature information is available in **Supplementary Materials 2**.

### 3.3 Radiomic models

#### 3.3.1 PT_Rad_models

**Figure 4:**
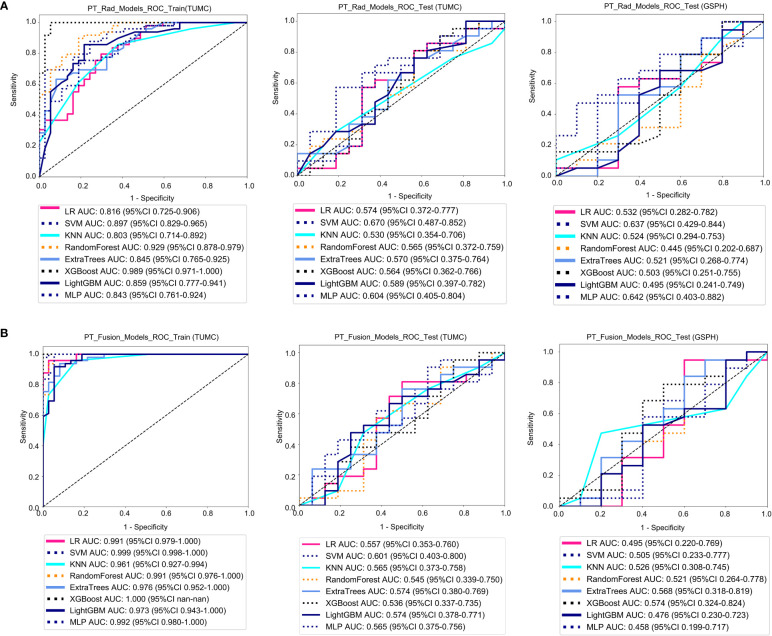
ROC curves for the ability of the radiomics models and fusion (radiomics and DTL) models to predict LLN metastasis in the training and validation cohorts. **(A)** Radiomics models based on PT. **(B)** Fusion models based on PT. (PT, primary tumor; PT_Rad_Models, radiomics models based on primary tumor; PT_Fusion_Models, the models combine radiomics and deep transfer learning features based on the primary tumor; ROC, receiver operating characteristic; RF, random forest; KNN, k-nearest neighbor; LR, logistic regression; MLP, multilayer perceptron; SVM, support vector machine; XGBoost, extreme gradient boosting; LightGBM, light gradient boosting machine; TUMC, Tianjin Union Medical Center; GSPH, Gansu Provincial Hospital).

**Figure 4A** shows the ROC analysis of radiomic features by different models in the training cohort and testing cohort. For the training cohort (TUMC), the AUC values for the LR, SVM, KNN, Random Forest, Extra Trees, XGBoost, LightGBM, and MLP models were 0.816, 0.897, 0.803, 0.929, 0.845, 0.989, 0.859, and 0.843, respectively. For the testing cohort (TUMC), the AUC values were 0.574, 0.670, 0.530, 0.566, 0.570, 0.564, 0.589, and 0.604, respectively. For the testing cohort (GSPH), the AUC values were 0.532, 0.637, 0.524, 0.445, 0.521, 0.503, 0.495, and 0.642, respectively. ​Detailed statistical evaluations of the PT_Rad_Models are presented in **Supplementary Table S2**. For a comparison of accuracy across different algorithms in the PT_Rad_Models, see **Supplementary Figure S6**. The confusion matrices for the training and test cohorts of the PT_Rad_Models are shown in **Supplementary Figure S12**. Waterfall plots for the training and test cohorts in the PT_Rad_Models can be found in **Supplementary Figure S18**. The results of the DCA for the training and test cohorts of the PT_Rad_Models are presented in **Supplementary Figure S24**.

#### 3.3.2 LLLN_Rad_models

**Figure 5:**
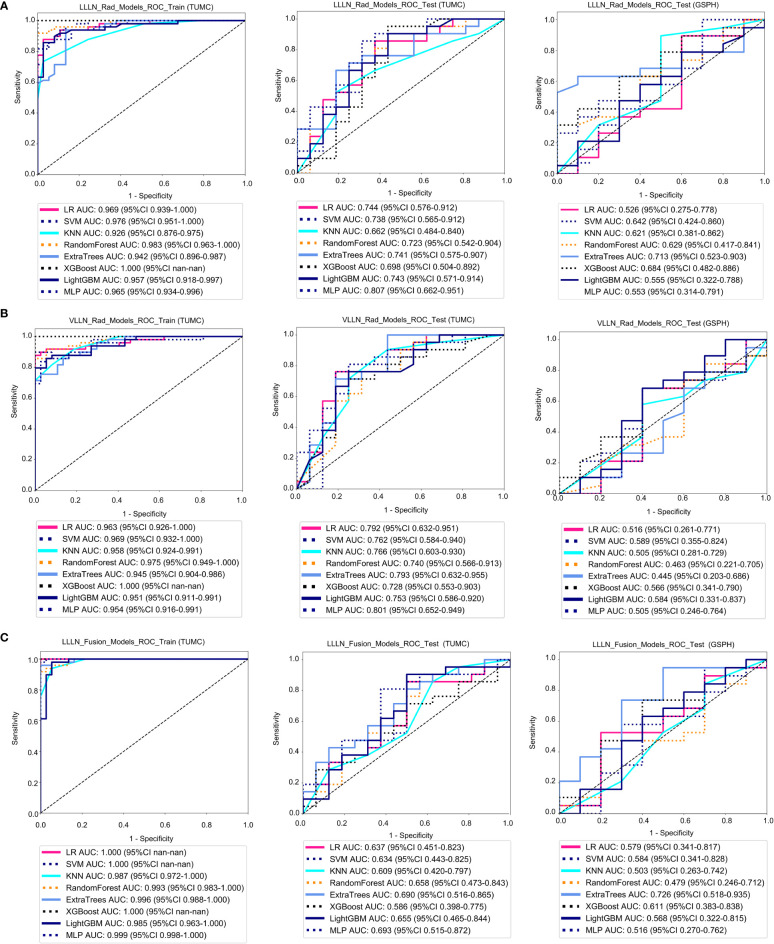
ROC curve for the radiomics models and fusion (radiomics and DTL) models to predict LLN metastasis in the training and validation cohorts. **(A)** Radiomics models based on LLLN. **(B)** Radiomics models based on VLLN. **(C)** Fusion models based on LLLN. (LLLN, largest short-axis lateral lymph node; VLLN, visible lateral lymph nodes; LLLN_Rad_Models, radiomics models based on largest short-axis lateral lymph node; LLLN_Fusion_Models, the models combine radiomics and deep transfer learning features based on largest short-axis lateral lymph node; ROC, receiver operating characteristic; RF, random forest; KNN, k-nearest neighbor; LR, logistic regression; MLP, multilayer perceptron; SVM, support vector machine; XGBoost, extreme gradient boosting; LightGBM, light gradient boosting machine; TUMC, Tianjin Union Medical Center; GSPH, Gansu Provincial Hospital).

**Figure 5A** shows the ROC analysis of radiomic features by different models in the training cohort and testing cohort. For the training cohort (TUMC), the AUC values for the LR, SVM, KNN, random forest, Extra Trees, XGBoost, LightGBM, and MLP models were 0.969, 0.976, 0.926, 0.983, 0.942, 1.000, 0.957, and 0.965, respectively. For the testing cohort (TUMC), the AUC values were 0.744, 0.738, 0.662, 0.723, 0.741, 0.698, 0.743, and 0.807, respectively. For the testing cohort (GSPH), the AUC values were 0.526, 0.642, 0.621, 0.629, 0.713, 0.684, 0.555, and 0.553, respectively. Detailed statistical evaluations of the LLLN_Rad_Models are presented in **Supplementary Table S4**. For a comparison of accuracy across different algorithms in the LLLN_Rad_Models, see **Supplementary Figure S8**. The confusion matrices for the training and test cohorts of the LLLN_Rad_Models are shown in **Supplementary Figure S14**. Waterfall plots for the training and test cohorts in the LLLN_Rad_Models can be found in **Supplementary Figure S20**. DCA for the training and test cohorts of the LLLN_Rad_Models is presented in **Supplementary Figure S26**.

#### 3.3.3 VLLN_Rad_models

**Figure 5B** shows the ROC analysis of radiomic features by different models in the training cohort and testing cohort. For the training cohort (TUMC), the AUC values for the LR, SVM, KNN, random forest, Extra Trees, XGBoost, LightGBM, and MLP models were 0.963, 0.969, 0.958, 0.975, 0.945, 1.000, 0.951, and 0.954, respectively. For the testing cohort (TUMC), the AUC values were 0.792, 0.762, 0.766, 0.740, 0.793, 0.728, 0.753, and 0.801, respectively. For the testing cohort (GSPH), the AUC values were 0.516, 0.589, 0.505, 0.463, 0.445, 0.566, 0.584, and 0.505, respectively. Detailed statistical evaluations of the VLLN_Rad_Models are presented in **Supplementary Table S5**. For a comparison of accuracy across different algorithms in the VLLN_Rad_Models, see **Supplementary Figure S9**. The confusion matrices for the training and test cohorts of the VLLN_Rad_Models are shown in **Supplementary Figure S15**. Waterfall plots for the training and test cohorts in the VLLN_Rad_Models can be found in **Supplementary Figure S21**. DCA for the training and test cohorts of the LLLN_Rad_Models is presented in **Supplementary Figure S27**.

**Figure 6:**
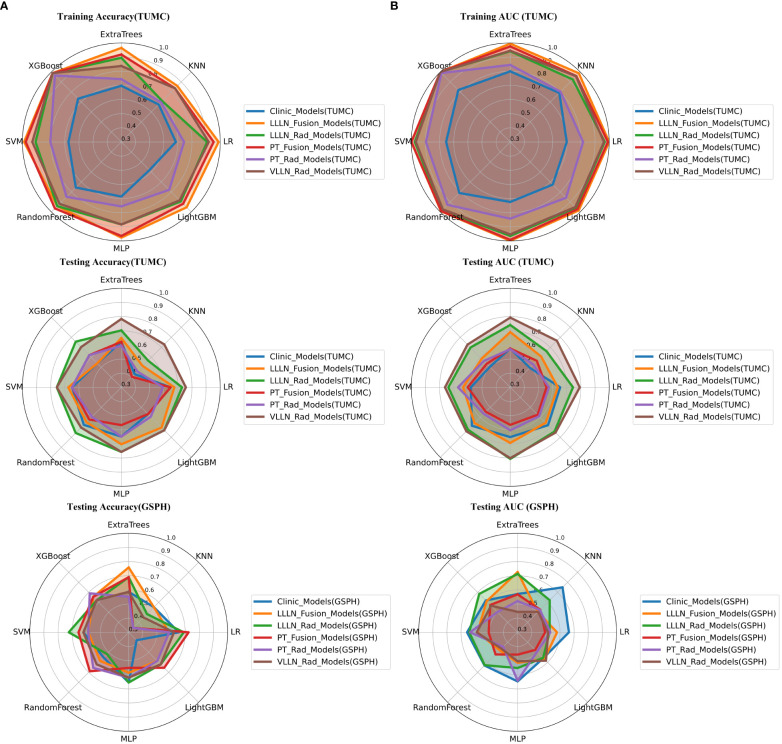
Radar chart of the accuracy and AUC of the models. **(A)** Accuracy. **(B)** AUC. (PT, primary tumor; LLLN, largest short-axis lateral lymph node; VLLN, visible lateral lymph nodes; PT_Rad_Models, radiomics models based on primary tumor; PT_Fusion_Models, the models combine radiomics and deep transfer learning features based on the primary tumor; LLLN_Rad_Models, radiomics models based on largest short-axis lateral lymph node; VLLN_Rad_Models, radiomics models based on all visible lateral lymph nodes; LLLN_Fusion_Models, the models combine radiomics and deep transfer learning features based on largest short-axis lateral lymph node; AUC, area under the curve; RF, random forest; KNN, k-nearest neighbor; LR, logistic regression; MLP, multilayer perceptron; SVM, support vector machine; XGBoost, extreme gradient boosting; LightGBM, light gradient boosting machine; TUMC, Tianjin Union Medical Center; GSPH, Gansu Provincial Hospital).

In terms of AUC, the LLLN_Rad_Models or VLLN_Rad_Models consistently performed better in the testing cohort (TUMC) than did the PT_Rad_Models across all models. In the testing cohort (GSPH), the classification ability of the VLLN_Rad_Models substantially decreased in terms of AUC, and the LLLN_Rad_Models also decreased, but to a lesser extent compared to the VLLN_Rad_Models. (**Figure 6**, **Supplementary Figure S29**).

### 3.4 Fusion models

#### 3.4.1 PT_Fusion_Models

**Figure 4B** shows the ROC analysis of radiomic features by different models in the training cohort and testing cohort. For the training cohort (TUMC), the AUC values for the LR, SVM, KNN, random forest, Extra Trees, XGBoost, LightGBM, and MLP models were 0.991, 0.999, 0.961, 0.991, 0.976, 1.000, 0.973, and 0.992, respectively. For the testing cohort (TUMC), the AUC values were 0.557, 0.601, 0.565, 0.545, 0.574, 0.536, 0.574, and 0.565, respectively. For the testing cohort (GSPH), the AUC values were 0.495, 0.505, 0.526, 0.521, 0.568, 0.574, 0.476, and 0.458, respectively. Detailed statistical evaluations of the PT_Fusion_Models are presented in **Supplementary Table S3**. For a comparison of accuracy across different algorithms in the PT_Fusion_Models, see **Supplementary Figure S7**. The confusion matrices for the training and test cohorts of the PT_Fusion_Models are shown in **Supplementary Figure S13**. Waterfall plots for the training and test cohorts in the PT_Fusion_Models can be found in **Supplementary Figure S19**. The results of the DCA for the training and test cohorts of the PT_Fusion_Models are presented in **Supplementary Figure S25**.

#### 3.4.2 LLLN_Fusion_Models

**Figure 5C** shows the ROC analysis of radiomic features by different models in the training cohort and testing cohort. For the training cohort (TUMC), the AUC values for the LR, SVM, KNN, random forest, Extra Trees, XGBoost, LightGBM, and MLP models were 1.000, 1.000, 0.987, 0.993, 0.996, 1.000, 0.985, and 0.999, respectively. For the testing cohort (TUMC), the AUC values were 0.637, 0.634, 0.609, 0.658, 0.690, 0.586, 0.655, and 0.693, respectively. For the testing cohort (GSPH, Gansu Provincial Hospital), the AUC values were 0.579, 0.584, 0.503, 0.479, 0.726, 0.611, 0.568, and 0.516, respectively. Detailed statistical evaluations of the LLLN_Fusion_Models are presented in **Supplementary Table S6**. For a comparison of accuracy across different algorithms in the LLLN_Fusion_Models, see **Supplementary Figure S10**. The confusion matrices for the training and test cohorts of the LLLN_Fusion_Models are shown in **Supplementary Figure S16**. Waterfall plots for the training and test cohorts in the LLLN_Fusion_Models can be found in **Supplementary Figure S22**. DCA for the training and test cohorts of the LLLN_Fusion_Models is presented in **Supplementary Figure S28**.

In the testing cohort (TUMC), LLLN_Fusion_Models outperformed the PT_Fusion_Models in terms of AUC for all algorithms. (**Figure 6**, **Supplementary Figure S30**).

### 3.5 Clinical models

**Figure 7:**
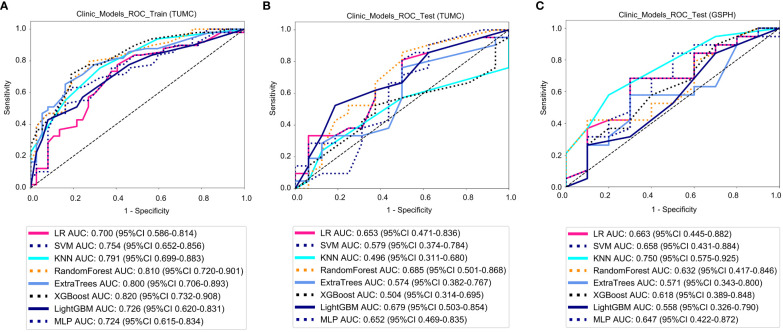
ROC curve for the clinical models for predicting LLN metastasis. **(A)** Clinical models in the training cohorts(TUMC). **(B)** Clinical models in the test cohorts (TUMC). **(C)** Clinical models in the test cohorts (GSPH, Gansu Provincial Hospital). (ROC, receiver operating characteristic; RF, random forest; KNN, k-nearest neighbor; LR, logistic regression; MLP, multilayer perceptron; SVM, support vector machine; XGBoost, extreme gradient boosting; LightGBM, light gradient boosting machine; TUMC, Tianjin Union Medical Center; GSPH, Gansu Provincial Hospital).

Clinical models: **Figure 7** shows the ROC analysis of clinical risk factors by different models in the training cohort and testing cohort. For the training cohort (TUMC), the AUC values for the LR, SVM, KNN, Random Forest, Extra Trees, XGBoost, LightGBM, and MLP models were 0.700, 0.754, 0.791, 0.810, 0.800, 0.820, 0.726, and 0.724, respectively. For the testing cohort (TUMC), the AUC values were 0.653, 0.579, 0.496, 0.685, 0.574, 0.504, 0.679, and 0.652, respectively. For the testing cohort (GSPH), the AUC values were 0.663, 0.658, 0.750, 0.632, 0.571, 0.618, 0.558, and 0.647, respectively.

Detailed statistical evaluations of the clinical models are presented in **Supplementary Table S1**. For a comparison of accuracy across different algorithms in the clinical models, see **Supplementary Figure S5**. The confusion matrices for the training and test cohorts of the clinical models are shown in **Supplementary Figure S11**. Waterfall plots for the training and test cohorts in the clinical models can be found in **Supplementary Figure S17**. DCA for the training and test cohorts of the clinical models is presented in **Supplementary Figure S23**.

### 3.6 DTL models

**Figure 8:**
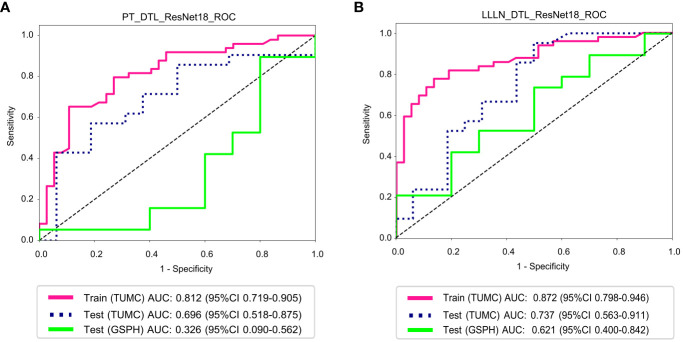
ROC curve for the DTL (ResNet18) models for predicting LLN metastasis in the training and validation cohorts. **(A)** PT_DTL_ ResNet18. **(B)** LLLN _DTL_ResNet18. (PT_DTL_ResNet18, deep transfer learning on primary tumor using ResNet18; LLLN_DTL_ResNet18, deep transfer learning on largest short-axis lateral lymph node using ResNet18; DTL, deep transfer learning; LLLN, largest short-axis lateral lymph node; PT, primary tumor; ROC, receiver operating characteristic; TUMC, Tianjin Union Medical Center; GSPH, Gansu Provincial Hospital).

**Figure 8** illustrates the ROC analysis for different DTL models in both the training and testing cohorts. PT_DTL_ResNet18 achieved an AUC of 0.812 in the training cohort, 0.696 in the testing cohort (TUMC) and 0.326 in the testing cohort (GSPH). Moreover, the AUC of the LLLN_DTL_ResNet18 model was 0.872 in the training cohort, 0.737 in the testing cohort (TUMC) and 0.621 in the testing cohort (GSPH, Gansu Provincial Hospital). The higher AUC values observed for LLLN_DTL_ResNet18 suggest that it may be a stronger model. Detailed statistical evaluations of these models are presented in **Table 2**.

**Table 2:** Model performance of the DTL (ResNet18) models.

Model name	Accuracy	AUC	95% CI	Sensitivity	Specificity	PPV	NPV	Precision	Recall	F1	Threshold	Task
PT_DTL_ResNet18	0.74	0.81	0.72-0.91	0.63	0.89	0.89	0.65	0.89	0.63	0.74	0.62	train(TUMC)
	0.65	0.70	0.52-0.88	0.52	0.81	0.79	0.57	0.79	0.52	0.63	0.64	test(TUMC)
	0.62	0.33	0.09-0.56	0.84	0.20	0.67	0.40	0.67	0.842	0.74	0.56	test(GSPH)
LLLN_DTL_ResNet18	0.80	0.87	0.80-0.95	0.76	0.87	0.88	0.73	0.88	0.76	0.81	0.58	train(TUMC)
	0.73	0.74	0.56-0.91	0.90	0.50	0.70	0.80	0.70	0.90	0.79	0.59	test(TUMC)
	0.62	0.62	0.40-0.84	0.68	0.50	0.72	0.45	0.72	0.48	0.70	0.76	test(GSPH)

PT_DTL_ResNet18, deep transfer learning on primary tumor using ResNet18; LLLN_DTL_ResNet18, deep transfer learning on largest short-axis lateral lymph node using ResNet18; AUC, area under the curve; PPV, positive predictive value; NPV, negative predictive value; F1, the harmonic mean of precision and recall; TUMC, Tianjin Union Medical Center; GSPH, Gansu Provincial Hospital.

## 4 Discussion

Lymph node metastasis in rectal cancer typically occurs in the mesorectum and LLNs. During the surgical treatment of rectal cancer, TME, which involves the routine removal of mesorectal lymph nodes, is commonly performed (14). Unlike mesorectal lymph nodes, LLNs are not typically included in the routine excision scope of TME. For patients suspected to have LLN metastasis, LLND is usually required to completely remove these metastatic LLNs (15).

Recent studies have indicated that for patients with rectal cancer diagnosed by imaging as having LLN metastasis, the postoperative pathologically positive concordance rates for LLND were 27.9 and 39.3%, respectively (16, 17). This suggests that in more than 60% of patients, LLND was unnecessary, as these patients endured the risks of surgery without oncological benefit. Thus, accurate preoperative diagnosis of LLN metastasis in rectal cancer patients is crucial, as the appropriateness of LLND directly determines its potential benefit to patients.

This study developed models based on DTL, radiomics, clinical, and fusion modeling for the prediction of LLN metastasis. Generally, all models showed superior performance in the training cohort compared to the testing cohort, indicating potential overfitting or the models’ inability to generalize well to unseen data. The PT_Rad_Models and PT_Fusion_Models performed poorly in both the testing set (TUMC) and the testing cohort (GSPH) (**Supplementary Figure S32**). The LLLN_Rad_Models consistently outperformed the PT_Rad_Models in AUC across all algorithmic implementations in the testing cohort (TUMC). Similarly, the LLLN_Fusion_Models consistently outperformed the PT_Fusion_Models in AUC across all algorithmic implementations in the testing cohort (TUMC). This might suggest that the radiomic features of the LLLN provide a more robust basis for model training and generalization than those of the PT. LLLN_DTL_ResNet18 showed better generalization from the training cohort (TUMC) to both testing cohorts (TUMC and GSPH) than PT_DTL_ResNet18. The consistently superior performance of LLLN_Rad_Models, LLLN_Fusion_Models and LLLN_DTL_ResNet18s in AUC in the testing cohort (TUMC) that LLLN data may provide a more robust and stable basis for predictions than PT data. Compared to PT data, models are more likely to learn patterns rather than noise from LLLN data.

Clinical models have a certain level of classification ability in the testing cohort, and this ability is less affected by the source of the testing data. In the external testing cohort (GSPH), the AUC for PT_DTL_ResNet18 was 0.326, while LLLN_DTL_ResNet18 still retained some classification ability with an AUC of 0.621. The possible reasons for this discrepancy could be the differences in scanning parameters between the two hospitals, leading to poor performance in the testing cohorts (GSPH). PT images are more susceptible to scanning parameter variations due to their dependency on imaging quality and contrast settings, whereas LLLN images provide more consistent features and are less affected by such variations.

In the training set, LLLN_Fusion_Models exhibited high AUC values, indicating a good fit to the training data. In contrast, LLLN_Rad_Models have lower AUC in training. However, in the testing set (TUMC), the LLLN_Fusion_Models did not perform better than the LLLN_Rad_Models for all algorithms (**Supplementary Figure S32**). This suggests that within the methodological framework used in this study, a richer feature pool does not enhance the models’ predictive efficacy on new datasets. The integration of a larger number of features might lead to models that perform well on training data but fail to generalize to new, unseen data. This can result from models capturing noise rather than underlying patterns.

Many machine learning studies on lymph node metastasis diagnosis in rectal cancer do not differentiate between mesorectal and LLNs (18–23). As a result, the models can only predict whether lymph node metastasis is present in patients but cannot determine whether metastasis occurs in the mesorectum or LLNs. This limitation restricts the clinical applicability of the models. There are a few focused studies attempting to address this issue. Yan H and colleagues constructed a diagnostic model for LLN metastasis based on clinical risk factors and radiomic features from MR images of primary rectal tumors and LLNs, achieving an AUC of 0.836 (24). Similarly, Yang H and others developed a model based on radiomic features from MR and CT images of LLNs combined with clinical risk factors, achieving an AUC of 0.936 (25). These studies segmented all VLLL, extracting 112 radiomic features from each VLLL. The maximum, minimum, mean, median, and standard deviation of each feature across all visible LLNs of each participant were recorded and analyzed using logistic regression. These studies did not perform external validation. Our research increased the number of extracted features to 1198, incorporated fusion models and DTL models, and included external testing cohorts. In terms of AUC, our findings show that while VLLN_Rad_Models outperformed LLLN_Rad_Models in the internal testing cohort (TUMC), their classification ability markedly declined in the external testing cohort (GSPH), making them less effective than LLLN_Rad_Models. This may be because the features of a single largest lateral lymph node are more stable and less affected by variations in scanning parameters and image quality. Handling features of a single lymph node also simplifies the model, reducing the risk of overfitting.

There are several limitations to this study. First, the relatively small sample size may limit the robustness of the results. Further multicenter studies with larger sample sizes are required to improve the diagnostic accuracy of the model and to validate its generalizability in predicting the pathological characteristics of LLN in rectal cancer patients prior to nCRT or surgery. Second, this study included patients who received nCRT before LLND, and only those with postoperative LLN pathology confirmed as positive were included. It is assumed that LLN metastasis occurred before nCRT and did not develop during treatment. This assumption might lead to bias in the results, as it does not consider the possibility that LLN metastasis could occur during nCRT, thereby affecting the accuracy and applicability of the predictive model based on pre-nCRT data.

## 5 Conclusion

This study demonstrated the diagnostic potential of radiomic, deep transfer learning, and fusion models for predicting LLN metastasis in rectal cancer patients. The use of LLLN data proved to be a more reliable basis for model prediction than PT data. While the fusion models showed high AUC values in the training set, they did not outperform the radiomic models when applied to unseen data. Among models performing adequately on the internal test set, all showed declines on the external test set, with LLLN_Rad_Models for diagnosing LLN metastasis being less affected by scanning parameters and data sources compared to other models.

## Data availability statement

The original contributions presented in the study are included in the article/**Supplementary Material**. Further inquiries can be directed to the corresponding author.

## Ethics statement

The studies involving humans were approved by Tianjin Union Medical Center’s Ethics Committee and Gansu Provincial Hospital's Ethics Committee. The studies were conducted in accordance with the local legislation and institutional requirements. Written informed consent for participation was not required from the participants or the participants’ legal guardians/next of kin in accordance with the national legislation and institutional requirements.

## Author contributions

YS: Visualization, Writing – original draft. ZL: Data curation, Writing – review & editing. HY: Validation, Visualization, Writing – original draft. PJ: Data curation, Writing – original draft. ZZ: Visualization, Writing – review & editing. JL: Data curation, Writing – original draft. YZ: Investigation, Writing – review & editing. PL: Validation, Writing – review & editing. QZ: Visualization, Writing – original draft. YL: Data curation, Writing – review & editing. LL: Data curation, Writing – review & editing. BD: Data curation, Writing – review & editing. XZ: Project administration, Supervision, Writing – review & editing.
