# Supplementary material for: Prediction of lateral lymph node metastasis in rectal cancer patients based on MRI using clinical, deep transfer learning, radiomic, and fusion models
Source: Front Oncol. 2024 Jul 19;14:1433190. doi: 10.3389/fonc.2024.1433190 (PMC11294238; doi:10.3389/fonc.2024.1433190)
Supplement: Supplementary file 1 [file DataSheet_1.pdf]

Supplementary materials

Prediction of Lateral Lymph Node Metastasis in Rectal Cancer Based on MRI Imaging Using, Clinical, Deep Transfer Learning, Radiomics, and Fusion Models

Supplementary Figures

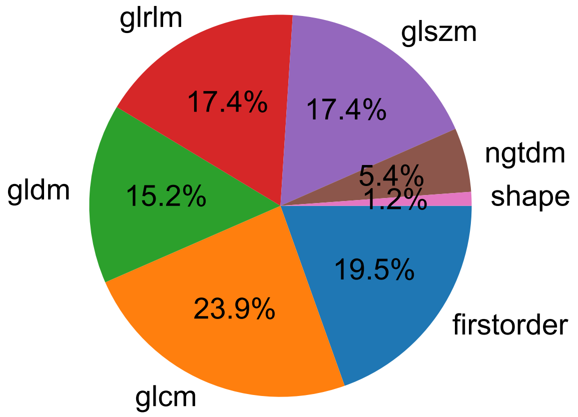

Supplementary Figure S1 The proportion, distribution and p of various radiomics features. (glcm, gray-level co-occurrence matrix; gldm, gray level dependence matrix; glrlm, gray-level run length matrix; glszm, gray-level size zone matrix; ngtdm, neighborhood gray-tone difference matrix.)

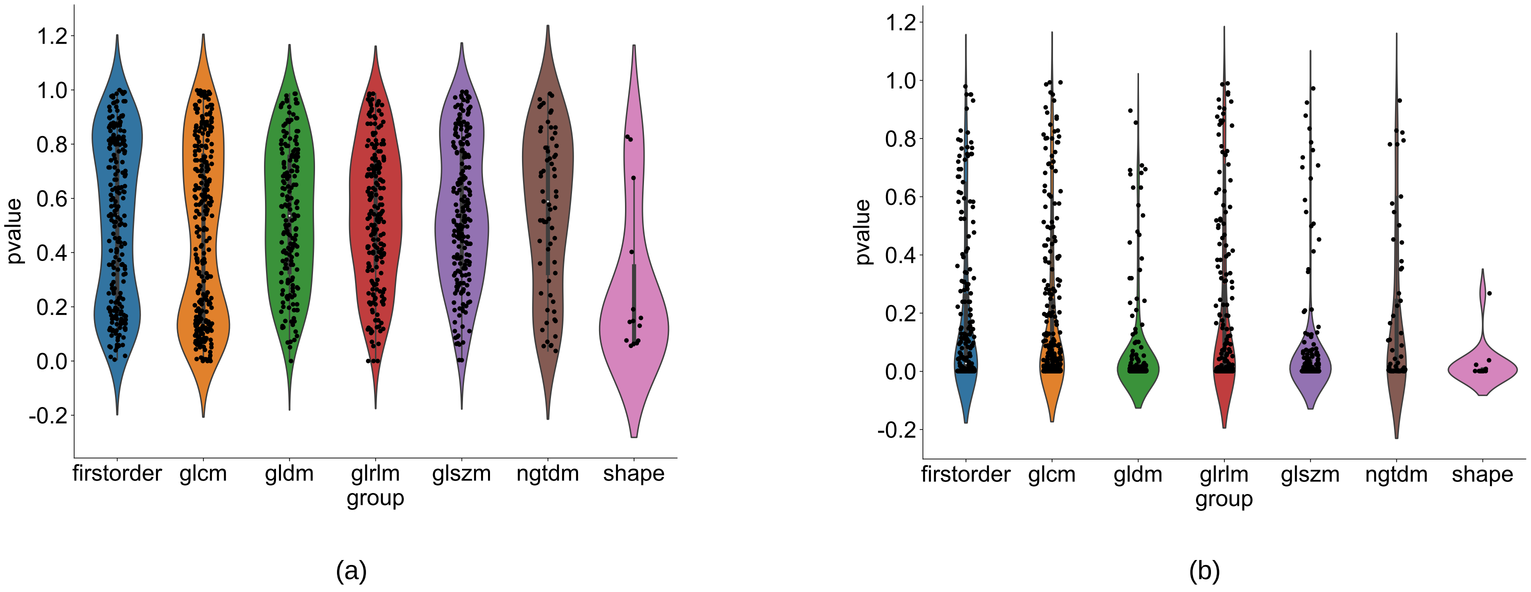

Supplementary Figure S2 The p-values of various radiomics features between the negative LLN metastasis group and the positive LLN metastasis group. (a) Features based on radiomics from PT. (b) Features based on radiomics from LLN. (PT, primary tumor; LLN, lateral lymph node; glcm, gray-level co-occurrence matrix; gldm, gray level dependence matrix; glrlm, gray-level run length matrix; glszm, gray-level size zone matrix; ngtdm, neighborhood gray-tone difference matrix.)

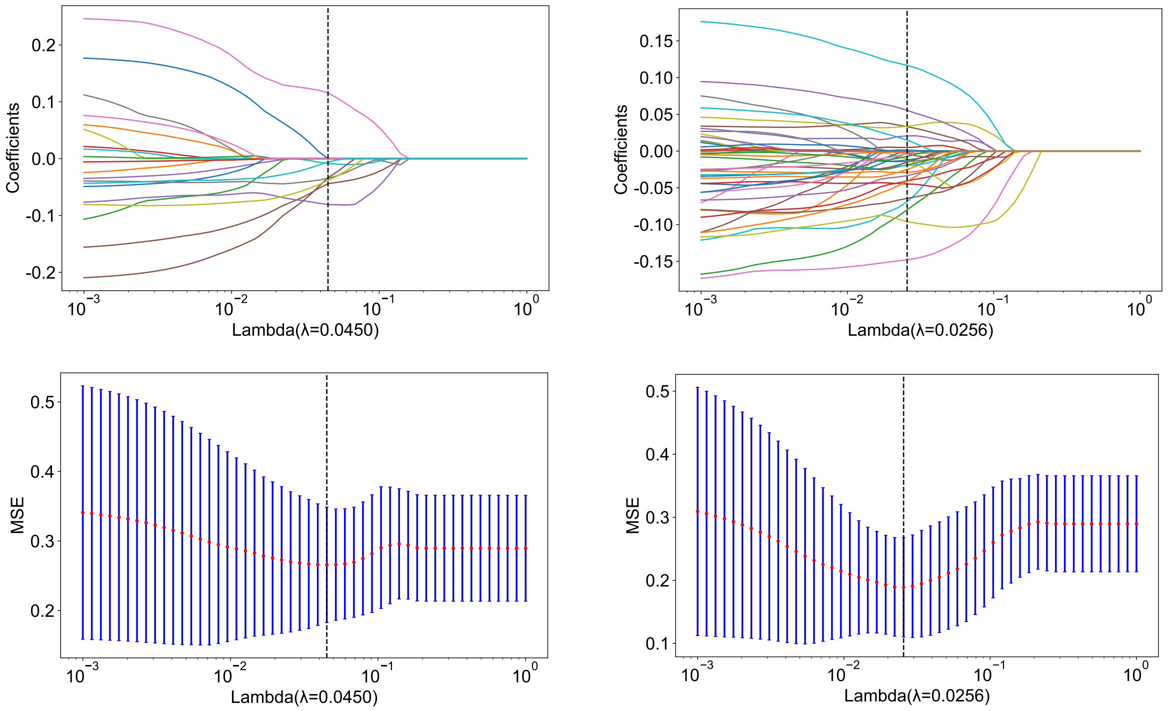

(a) (b)

Supplementary Figure S3. Feature selection based on LASSO algorithm. (a) Features based on radiomics from PT. （b）Features based on radiomics and DTL(ResNet18) from PT. (PT, primary tumor; DTL, deep transfer learning; MSE, Mean Squared Error.)

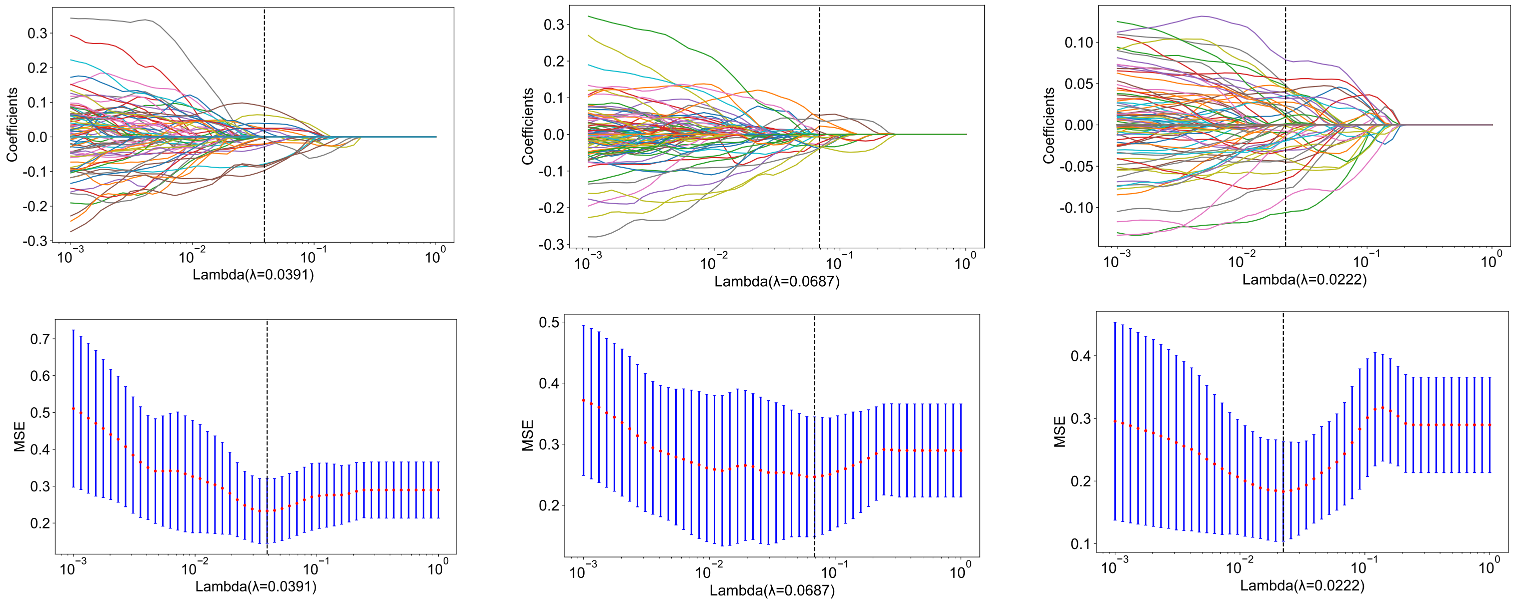

(a) (b) (c)

Supplementary Figure S4. Feature selection based on LASSO algorithm. (a) Features based on radiomics from LLLN. （b）Features based on radiomics from VLLN. （c）Features based on radiomics and DTL(ResNet18) from LLN. (LLLN, largest short-axis lateral lymph node; VLLN, visible lateral lymph nodes; DTL, deep transfer learning; MSE, Mean Squared Error.)

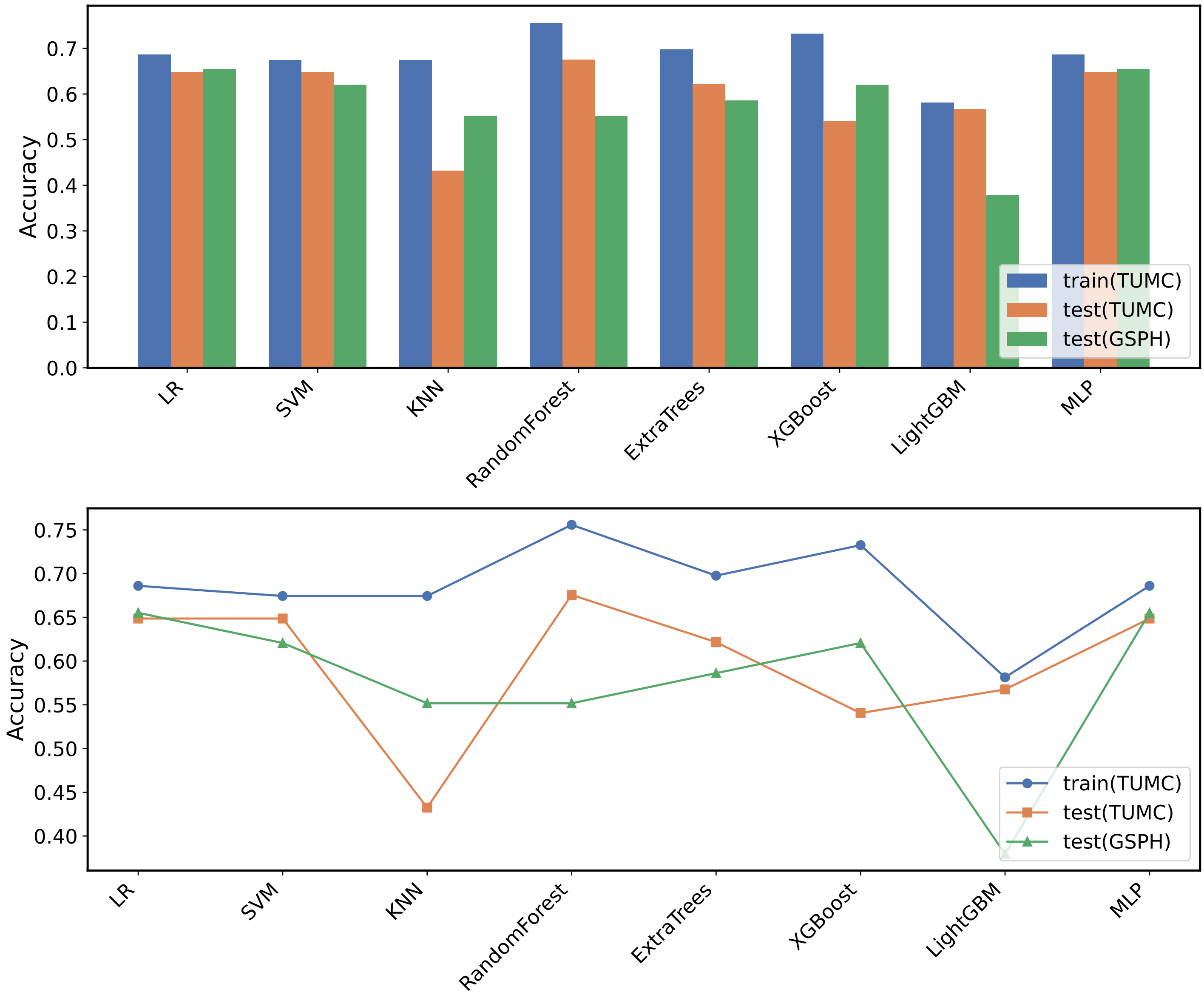

Supplementary Figure S5. Accuracy in clinical models. (RF, random forest; KNN, k-nearest neighbor; LR, logistic regression; MLP, multilayer perceptron; SVM, support vector machine; XGBoost, extreme gradient boosting; LightGBM, light gradient boosting machine; TUMC, Tianjin Union Medical Center; GSPH, Gansu Provincial Hospital.)

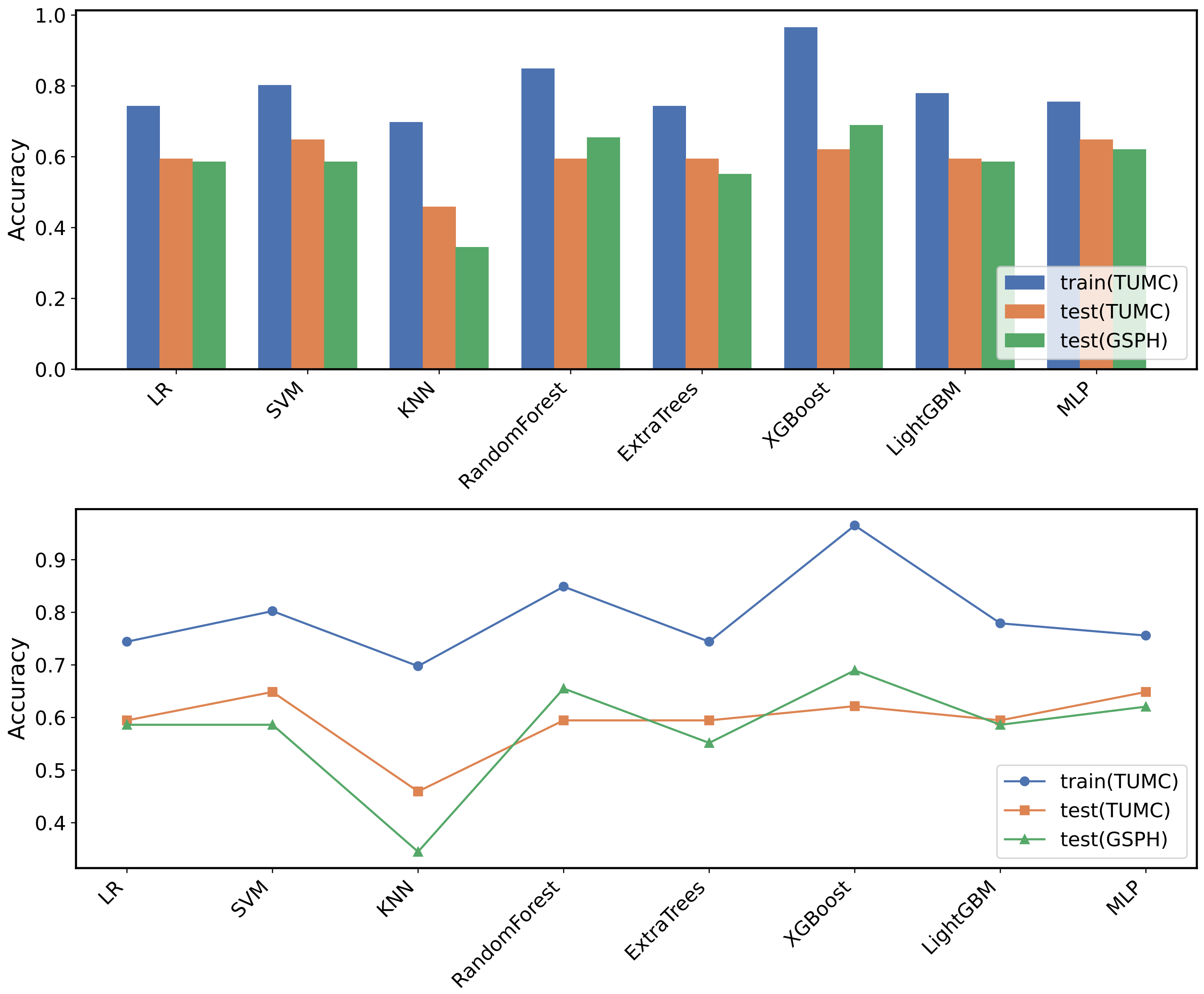

Supplementary Figure S6. Accuracy in PT_Rad_Models. (PT_Rad_Models, the radiomics models based on primary tumor; RF, random forest; KNN, k-nearest neighbor; LR, logistic regression; MLP, multilayer perceptron; SVM, support vector machine; XGBoost, extreme gradient boosting; LightGBM, light gradient boosting machine; TUMC, Tianjin Union Medical Center; GSPH, Gansu Provincial Hospital.)

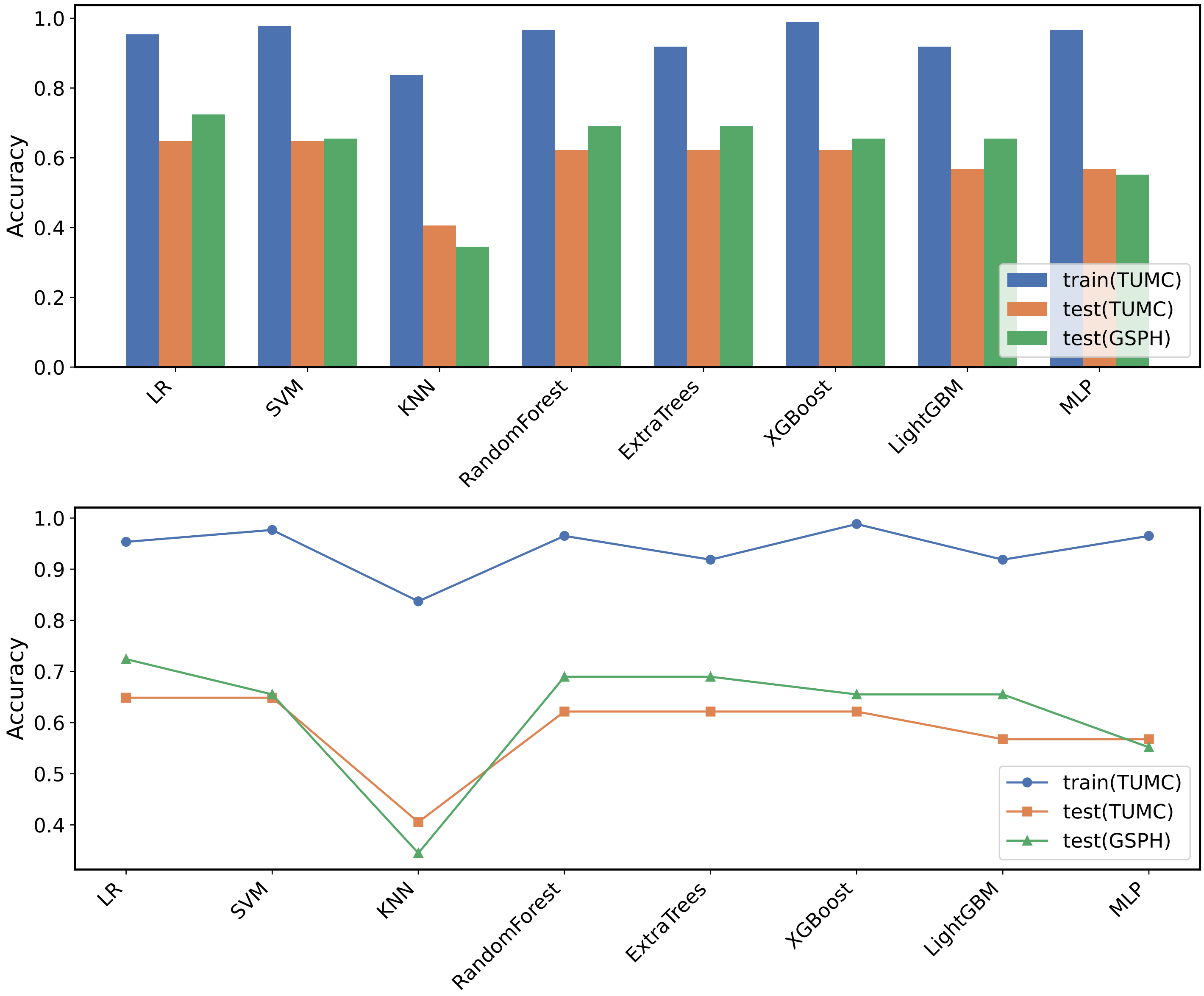

Supplementary Figure S7. Accuracy in PT_Fusion_Models. (PT_Fusion_Models, the models combine radiomics and deep transfer learning features based on the primary tumor; RF, random forest; KNN, k-nearest neighbor; LR, logistic regression; MLP, multilayer perceptron; SVM, support vector machine; XGBoost, extreme gradient boosting; LightGBM, light gradient boosting machine; TUMC, Tianjin Union Medical Center; GSPH, Gansu Provincial Hospital.)

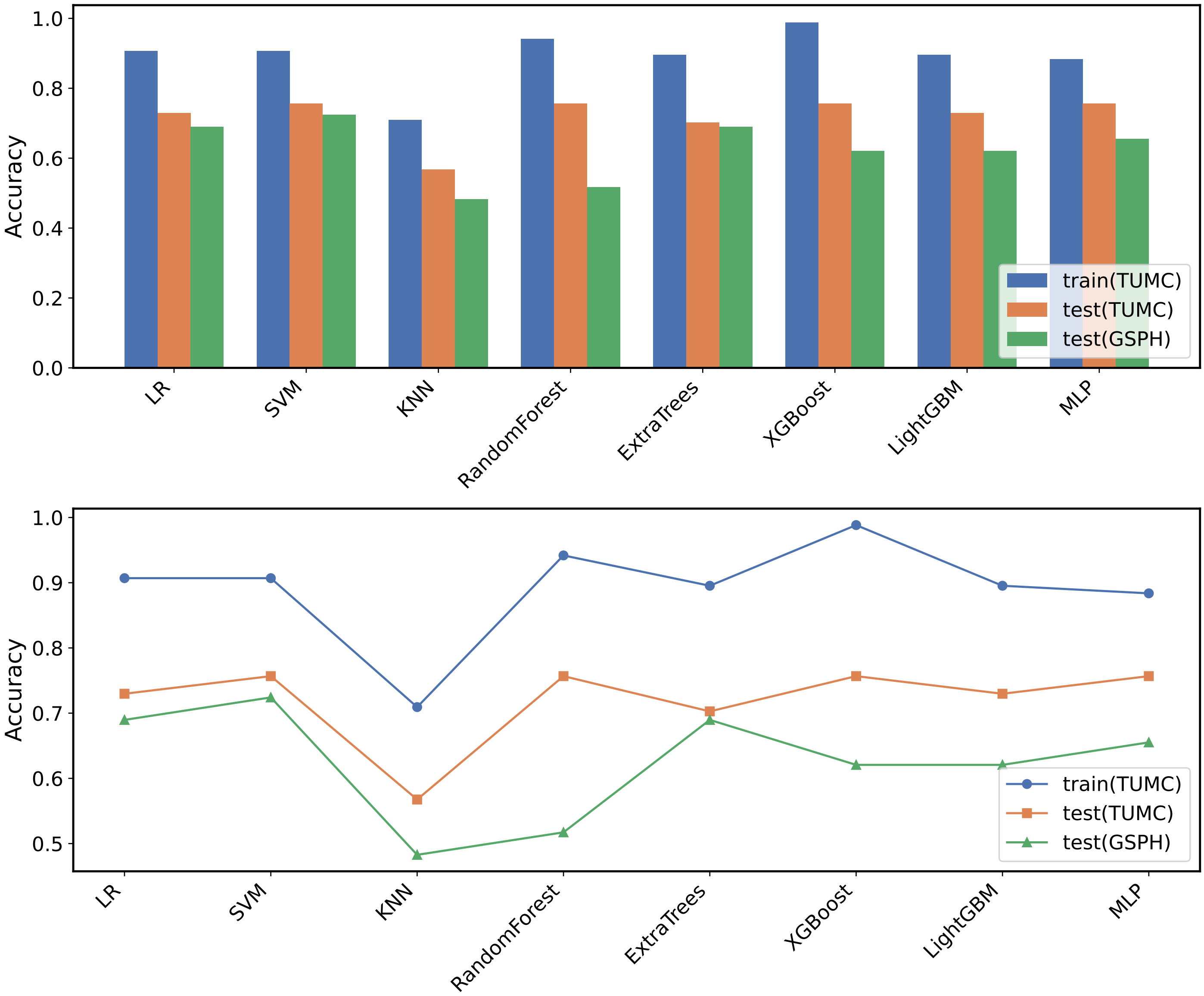

Supplementary Figure S8. Accuracy in LLLN_Rad_Models. (LLLN_Rad_Models, the radiomics model based on largest short-axis lateral lymph node; RF, random forest; KNN, k-nearest neighbor; LR, logistic regression; MLP, multilayer perceptron; SVM, support vector machine; XGBoost, extreme gradient boosting; LightGBM, light gradient boosting machine; TUMC, Tianjin Union Medical Center; GSPH, Gansu Provincial Hospital.)

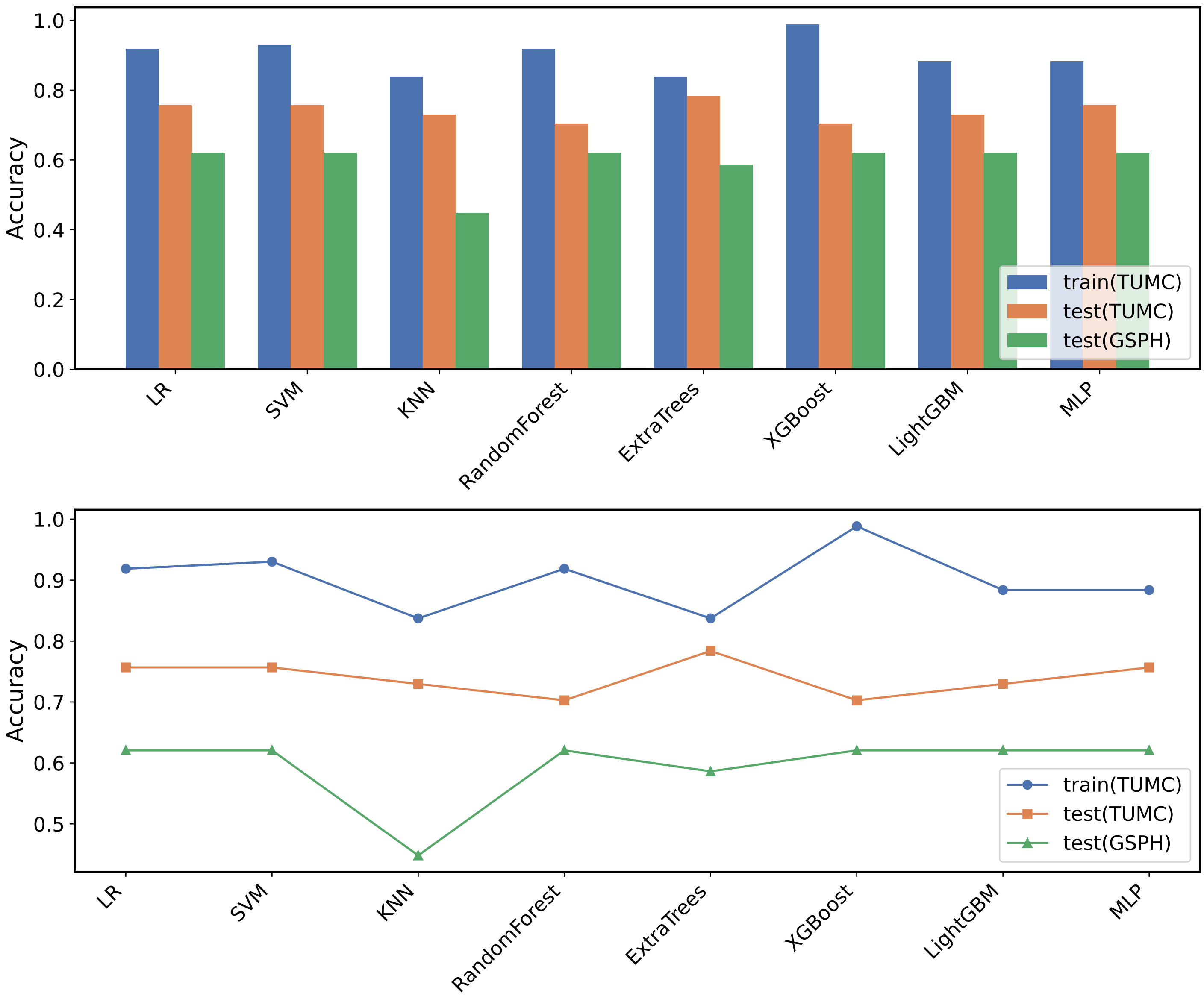

Supplementary Figure S9. Accuracy in VLLN_Rad_Models. (VLLN_Rad_Models, the radiomics model based on all visible lateral lymph nodes; DTL, deep transfer learning; RF, random forest; KNN, k-nearest neighbor; LR, logistic regression; MLP, multilayer perceptron; SVM, support vector machine; XGBoost, extreme gradient boosting; LightGBM, light gradient boosting machine; TUMC, Tianjin Union Medical Center; GSPH, Gansu Provincial Hospital.)

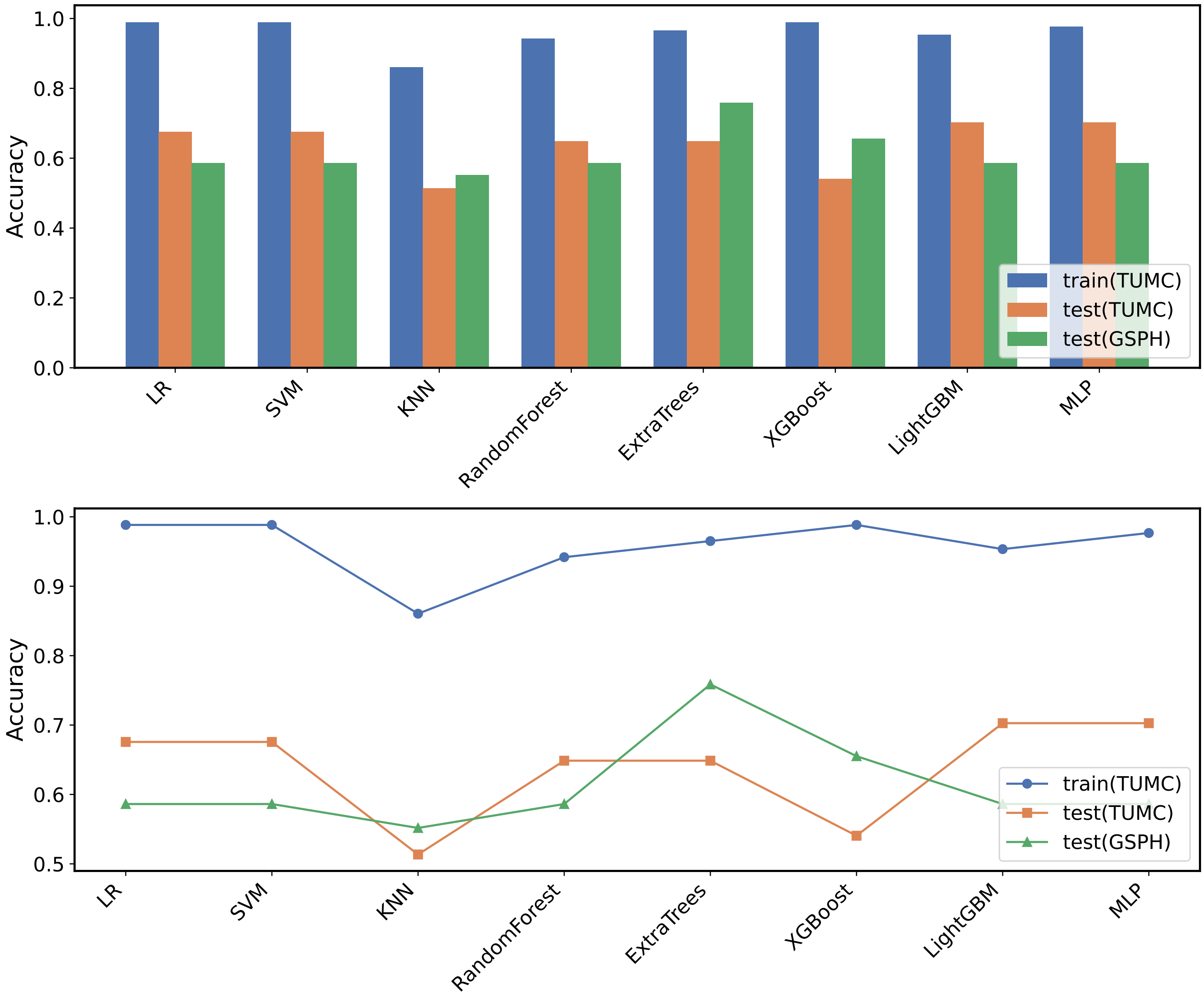

Supplementary Figure S10. Accuracy in LLLN_Fusion_Models. (LLLN_Fusion_Models, the models combine radiomics and deep transfer learning features based on largest short-axis lateral lymph node; DTL, deep transfer learning; RF, random forest; KNN, k-nearest neighbor; LR, logistic regression; MLP, multilayer perceptron; SVM, support vector machine; XGBoost, extreme gradient boosting; LightGBM, light gradient boosting machine; TUMC, Tianjin Union Medical Center; GSPH, Gansu Provincial Hospital.)

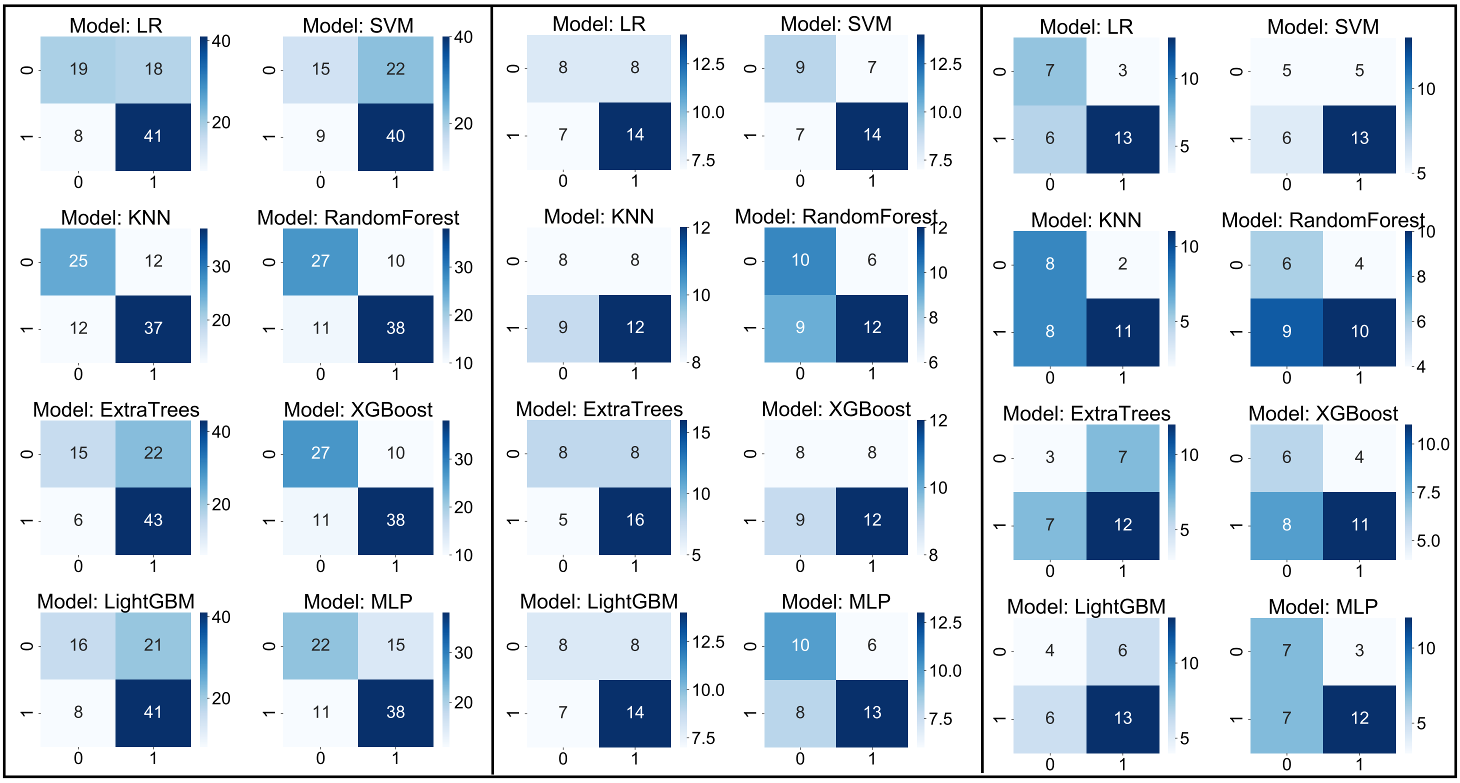

(a) (b) (c)

Supplementary Figure S11. Confusion matrix in clinical models. (a) Training cohort (TUMC). (b) Test cohort (TUMC). (c) Test cohort (GSPH). (RF, random forest; KNN, k-nearest neighbor; LR, logistic regression; MLP, multilayer perceptron; SVM, support vector machine; XGBoost, extreme gradient boosting; LightGBM, light gradient boosting machine; TUMC, Tianjin Union Medical Center; GSPH, Gansu Provincial Hospital.)

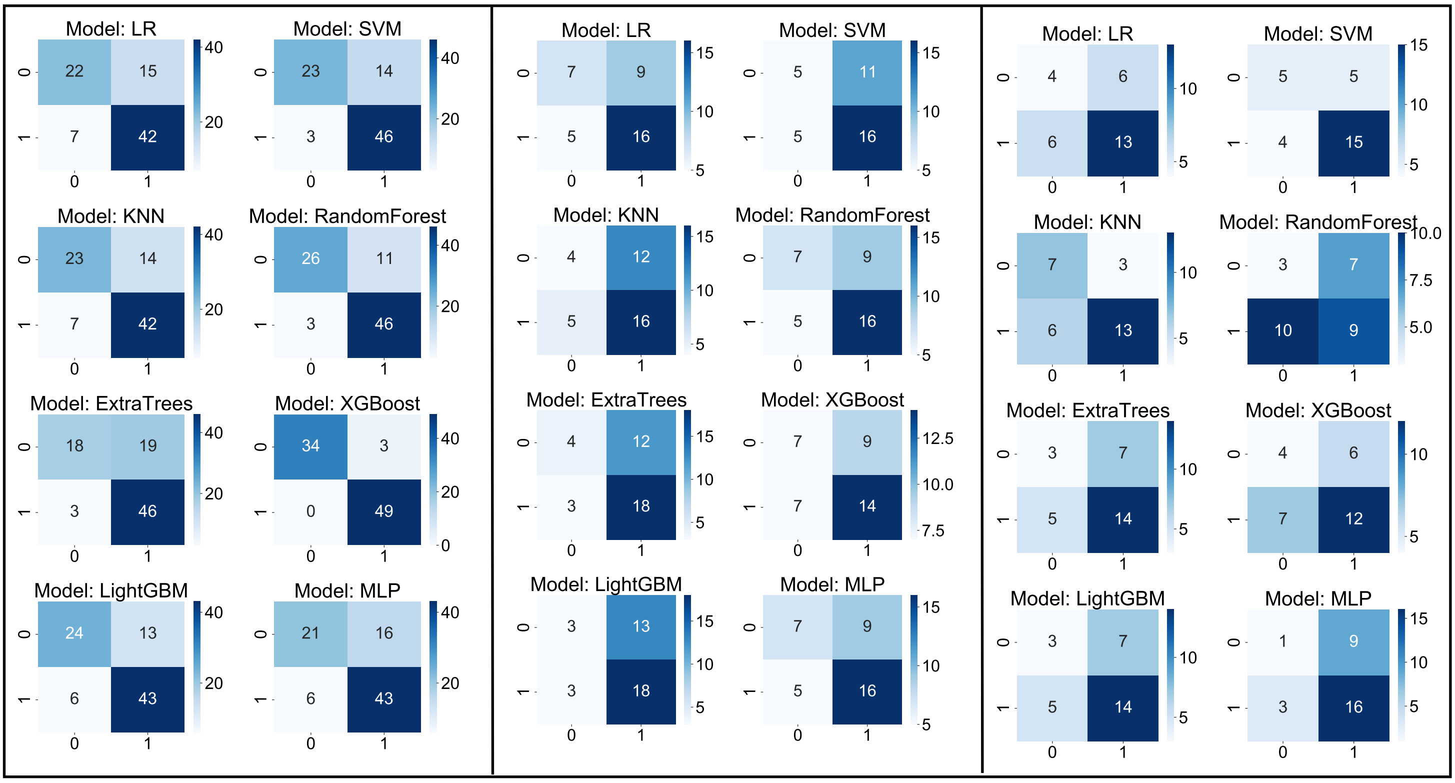

(a) (b) (c)

Supplementary Figure S12. Confusion matrix in PT_Rad_Models. (a) Training cohort (TUMC). (b) Test cohort (TUMC). (c) Test cohort (GSPH). (PT_Rad_Models, the radiomics models based on primary tumor; RF, random forest; KNN, k-nearest neighbor; LR, logistic regression; MLP, multilayer perceptron; SVM, support vector machine; XGBoost, extreme gradient boosting; LightGBM, light gradient boosting machine; TUMC, Tianjin Union Medical Center; GPPH, Gansu Provincial Hospital.)

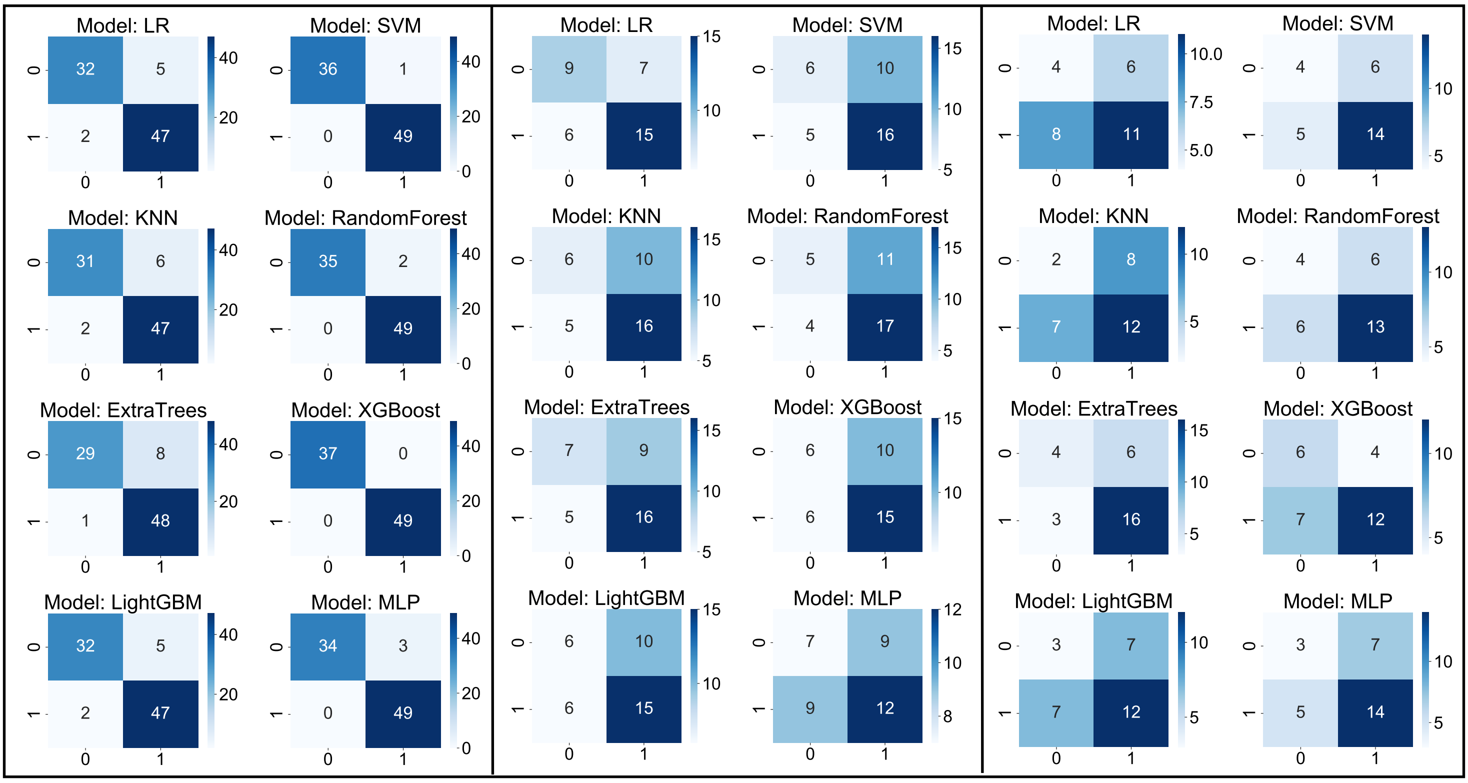

(a) (b) (c)

Supplementary Figure S13. Confusion matrix in PT_Fusion_Models. (a) Training cohort (TUMC). (b) Test cohort (TUMC). (c) Test cohort (GSPH). (PT_Fusion_Models, the models combine radiomics and deep transfer learning features based on the primary tumor; RF, random forest; KNN, k-nearest neighbor; LR, logistic regression; MLP, multilayer perceptron; SVM, support vector machine; XGBoost, extreme gradient boosting; LightGBM, light gradient boosting machine; TUMC, Tianjin Union Medical Center; GSPH, Gansu Provincial Hospital.)

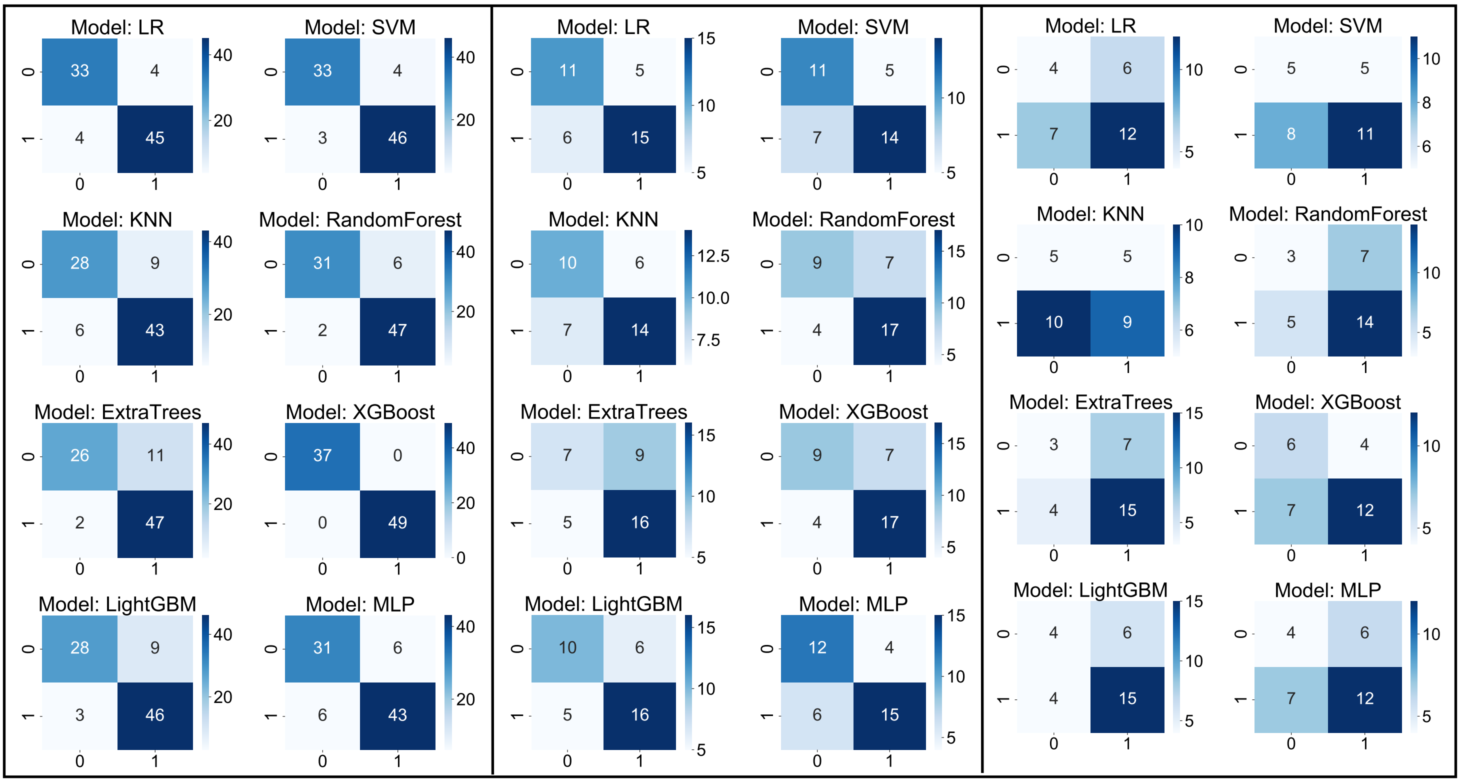

Supplementary Figure S14. Confusion matrix in LLLN_Rad_Models. (a) Training cohort (TUMC). (b) Test cohort (TUMC). (c) Test cohort (GSPH). (LLLN_Rad_Models, the radiomics model based on largest short-axis lateral lymph node; RF, random forest; KNN, k-nearest neighbor; LR, logistic regression; MLP, multilayer perceptron; SVM, support vector machine; XGBoost, extreme gradient boosting; LightGBM, light gradient boosting machine; TUMC, Tianjin Union Medical Center; GSPH, Gansu Provincial Hospital.)

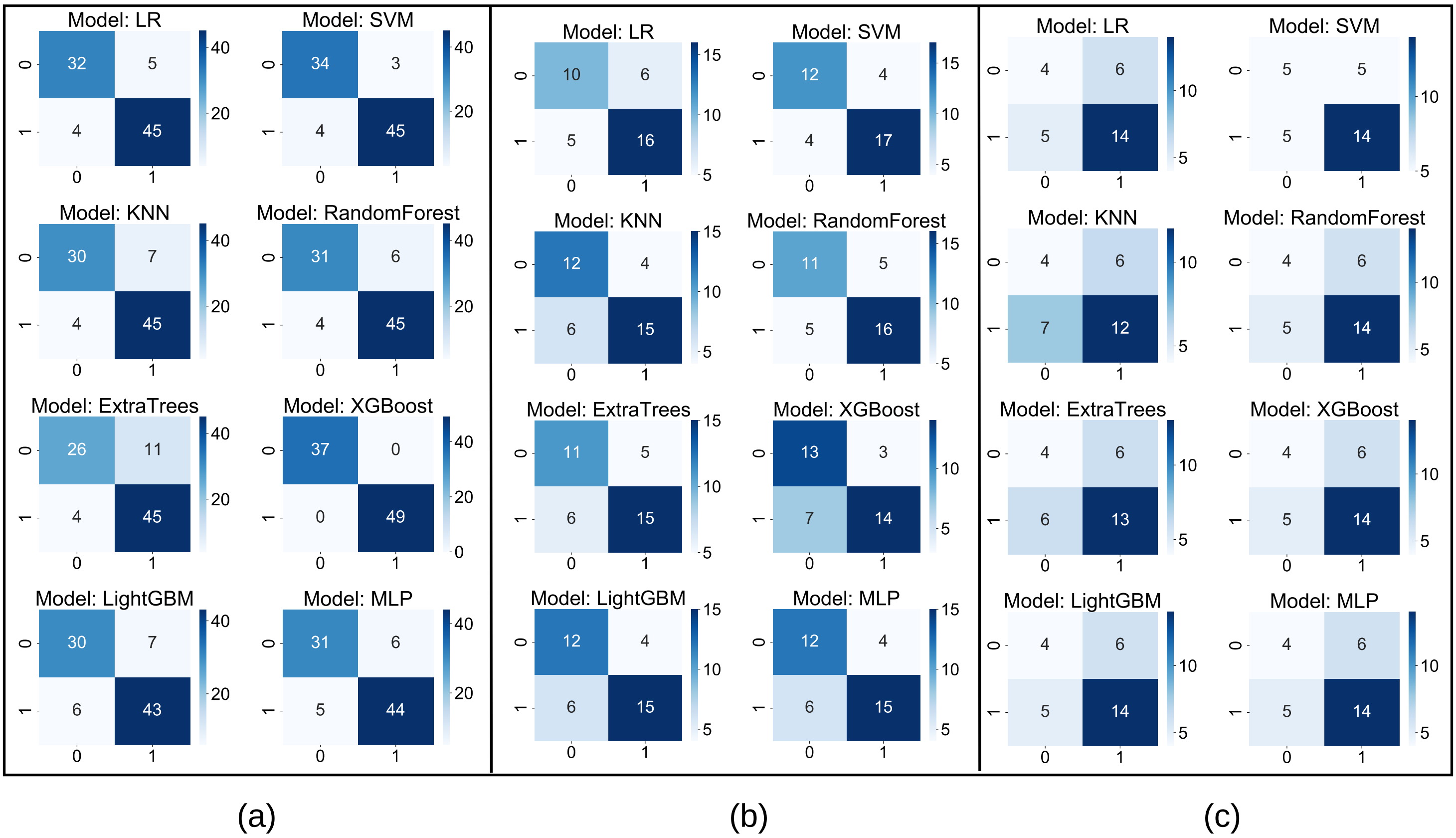

Supplementary Figure S15. Confusion matrix in VLLN_Rad_Models. (a) Training cohort (TUMC). (b) Test cohort (TUMC). (c) Test cohort (GSPH). (VLLN_Rad_Models, the radiomics model based on all visible lateral lymph nodes; RF, random forest; KNN, k-nearest neighbor; LR, logistic regression; MLP, multilayer perceptron; SVM, support vector machine; XGBoost, extreme gradient boosting; LightGBM, light gradient boosting machine; TUMC, Tianjin Union Medical Center; GSPH, Gansu Provincial Hospital.)

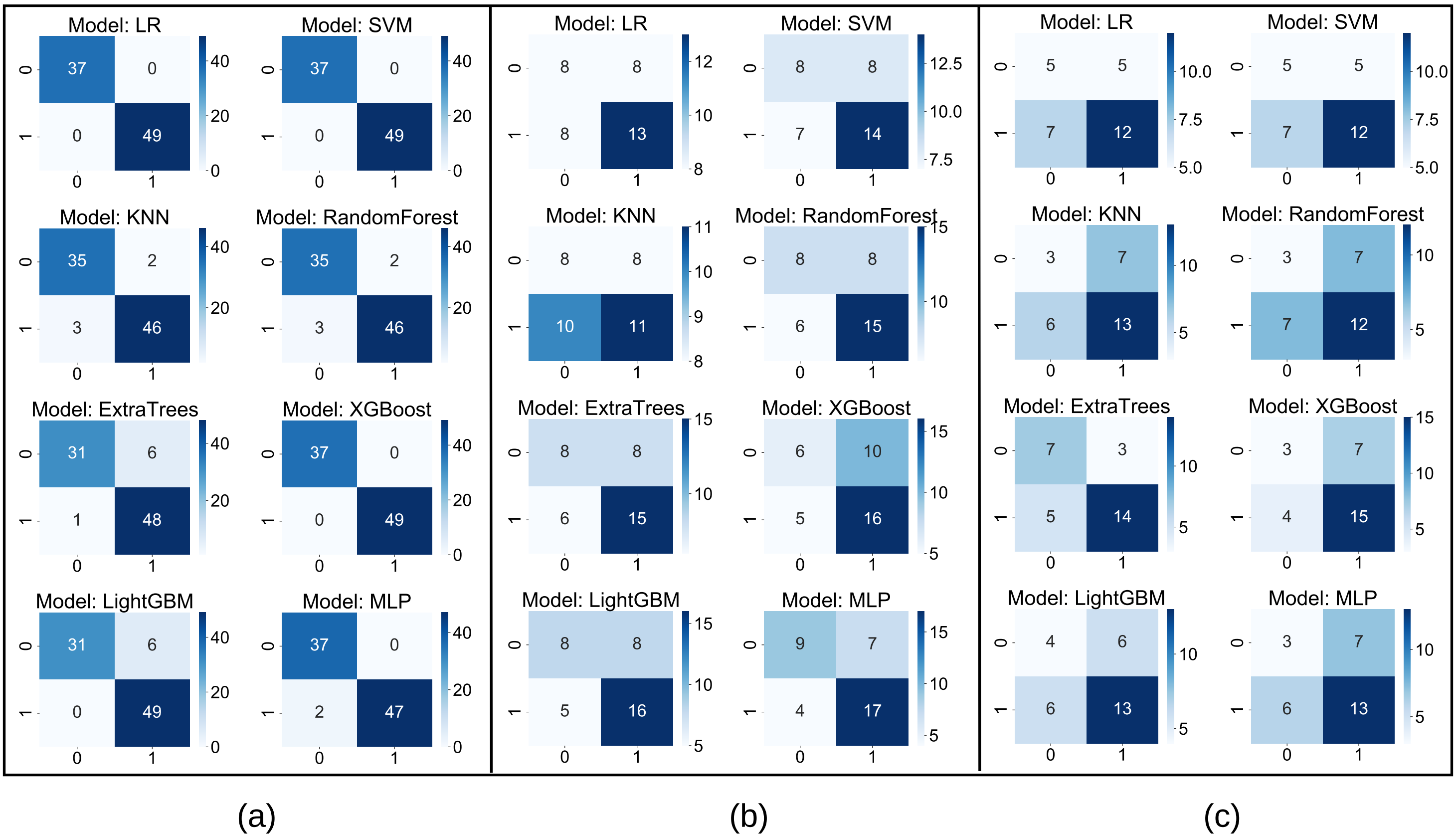

Supplementary Figure S16. Confusion matrix in LLLN_Fusion_Models. (a) Training cohort (TUMC). (b) Test cohort (TUMC). (c) Test cohort (GSPH). (LLLN_Fusion_Models, the models combine radiomics and deep transfer learning features based on largest short-axis lateral lymph node; RF, random forest; KNN, k-nearest neighbor; LR, logistic regression; MLP, multilayer perceptron; SVM, support vector machine; XGBoost, extreme gradient boosting; LightGBM, light gradient boosting machine; TUMC, Tianjin Union Medical Center; GSPH, Gansu Provincial Hospital.)

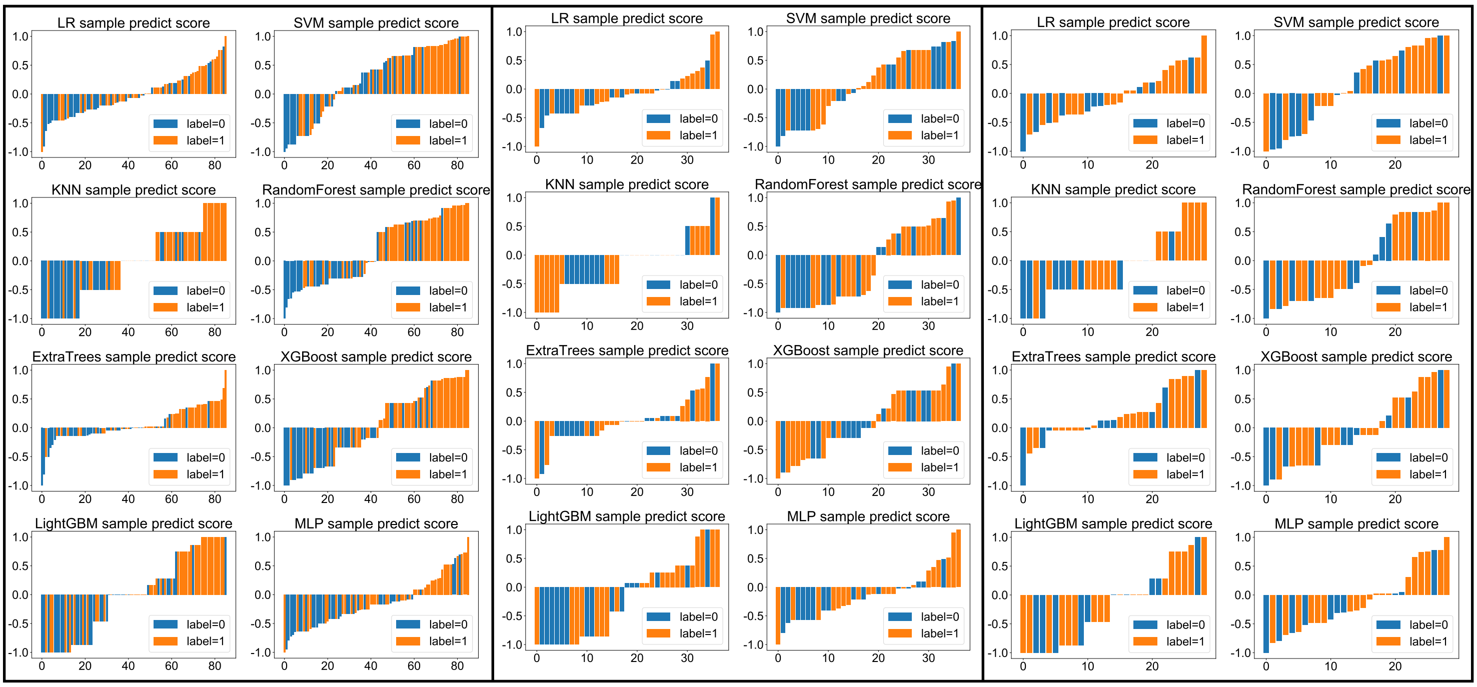

(a) (b) (c)

Supplementary Figure S17. Waterfall figure in clinical models. (a) Training cohort (TUMC). (b) Test cohort (TUMC). (c) Test cohort (GSPH). (RF, random forest; KNN, k-nearest neighbor; LR, logistic regression; MLP, multilayer perceptron; SVM, support vector machine; XGBoost, extreme gradient boosting; LightGBM, light gradient boosting machine; TUMC, Tianjin Union Medical Center; GSPH, Gansu Provincial Hospital.)

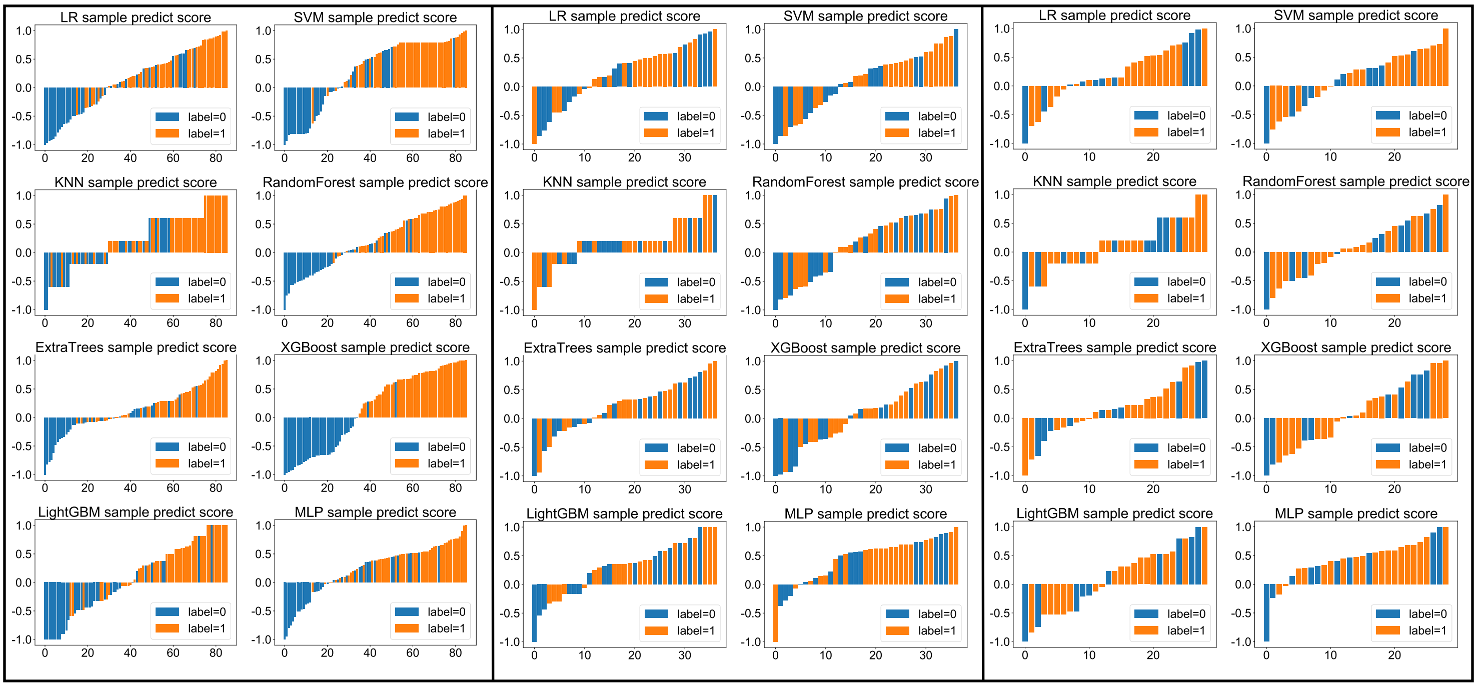

(a) (b) (c)

Supplementary Figure S18. Waterfall figure in PT_Rad_Models. (a) Training cohort (TUMC). (b) Test cohort (TUMC). (c) Test cohort (GSPH). (PT_Rad_Models, the radiomics models based on primary tumor; RF, random forest; KNN, k-nearest neighbor; LR, logistic regression; MLP, multilayer perceptron; SVM, support vector machine; XGBoost, extreme gradient boosting; LightGBM, light gradient boosting machine; TUMC, Tianjin Union Medical Center; GSPH, Gansu Provincial Hospital.)

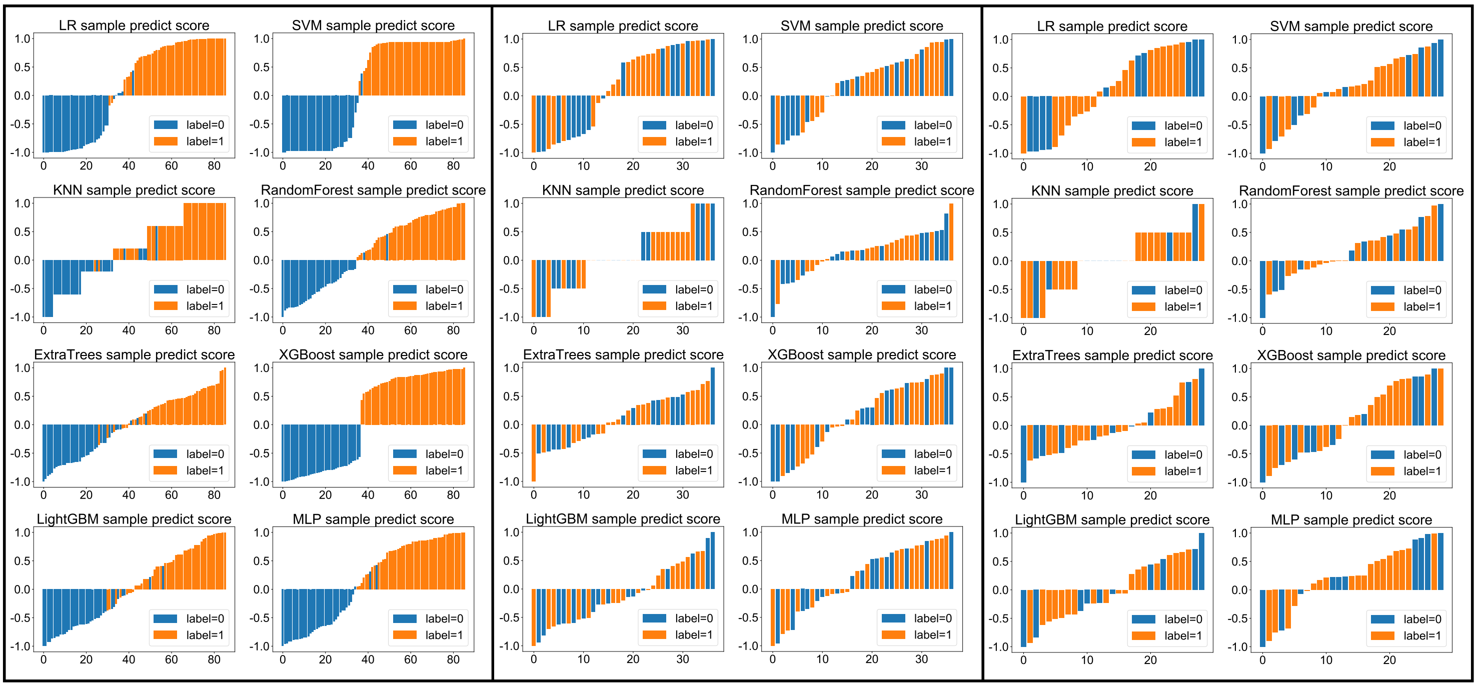

(a) (b) (c)

Supplementary Figure S19. Waterfall figure in PT_Fusion_Model. (a) Training cohort (TUMC). (b) Test cohort (TUMC). (c) Test cohort (GSPH). (PT_Fusion_Models, the models combine radiomics and deep transfer learning features based on the primary tumor; RF, random forest; KNN, k-nearest neighbor; LR, logistic regression; MLP, multilayer perceptron; SVM, support vector machine; XGBoost, extreme gradient boosting; LightGBM, light gradient boosting machine; TUMC, Tianjin Union Medical Center; GSPH, Gansu Provincial Hospital.)

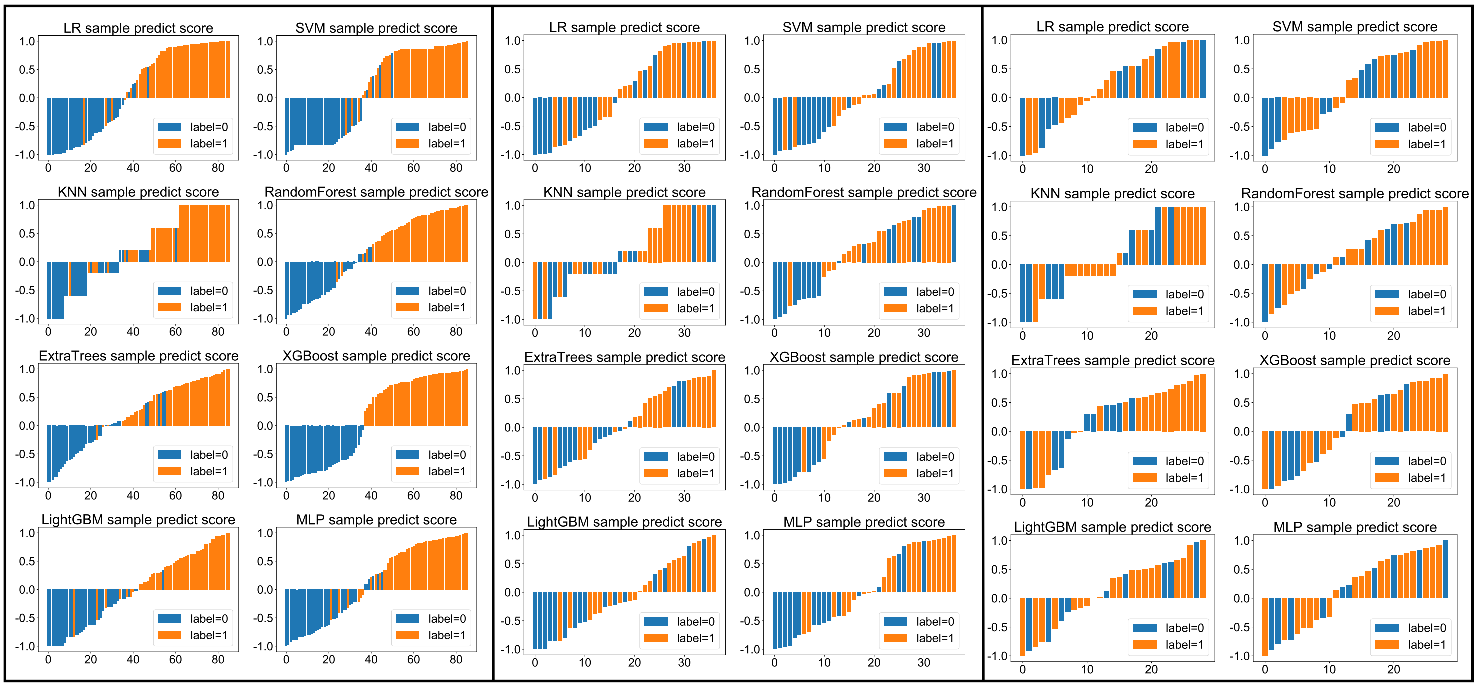

(a) (b) (c)

Supplementary Figure S20. Waterfall figure in LLLN_Rad_Models. (a) Training cohort (TUMC). (b) Test cohort (TUMC). (c) Test cohort (GSPH). (LLLN_Rad_Models, the radiomics model based on largest short-axis lateral lymph node; RF, random forest; KNN, k-nearest neighbor; LR, logistic regression; MLP, multilayer perceptron; SVM, support vector machine; XGBoost, extreme gradient boosting; LightGBM, light gradient boosting machine; TUMC, Tianjin Union Medical Center; GSPH, Gansu Provincial Hospital.)

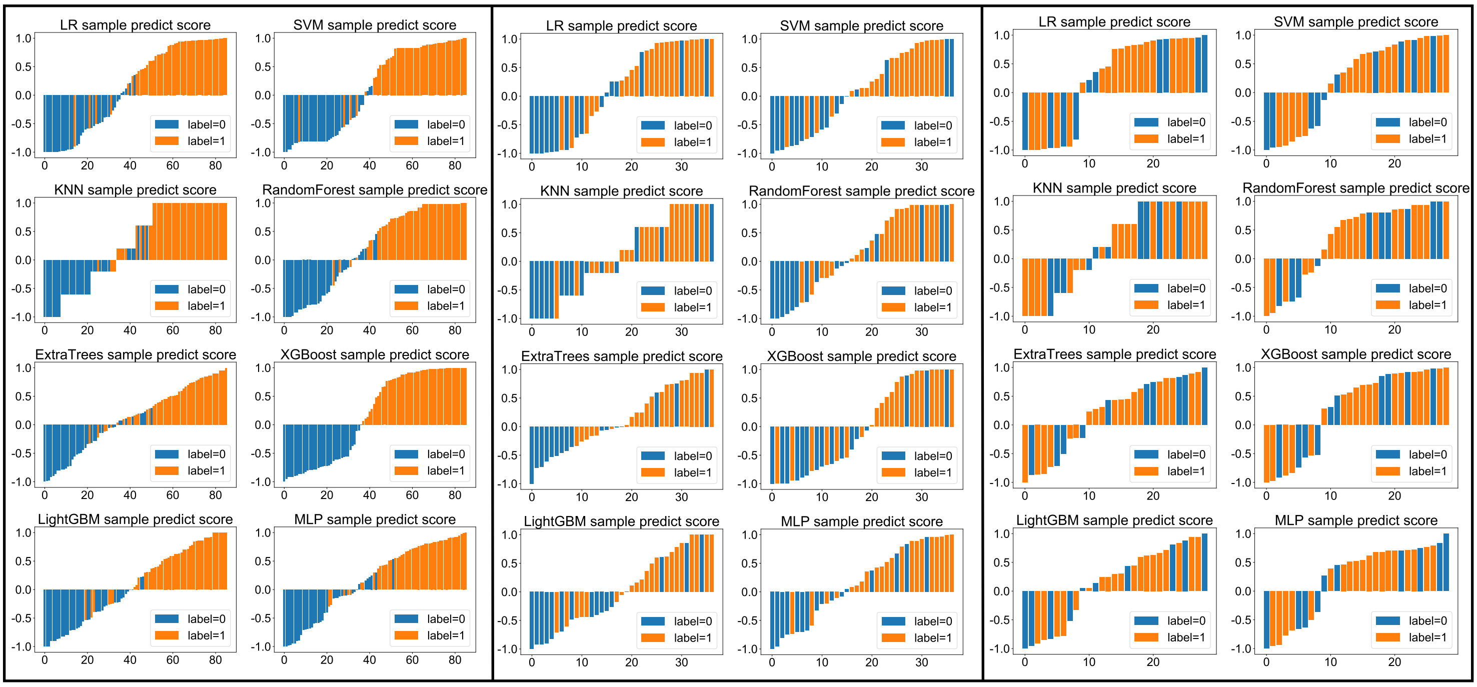

(a) (b) (c)

Supplementary Figure S21. Waterfall figure in VLLN_Rad_Models. (a) Training cohort (TUMC). (b) Test cohort (TUMC). (c) Test cohort (GSPH). (VLLN_Rad_Models, the radiomics model based on all visible lateral lymph nodes; RF, random forest; KNN, k-nearest neighbor; LR, logistic regression; MLP, multilayer perceptron; SVM, support vector machine; XGBoost, extreme gradient boosting; LightGBM, light gradient boosting machine; TUMC, Tianjin Union Medical Center; GSPH, Gansu Provincial Hospital.)

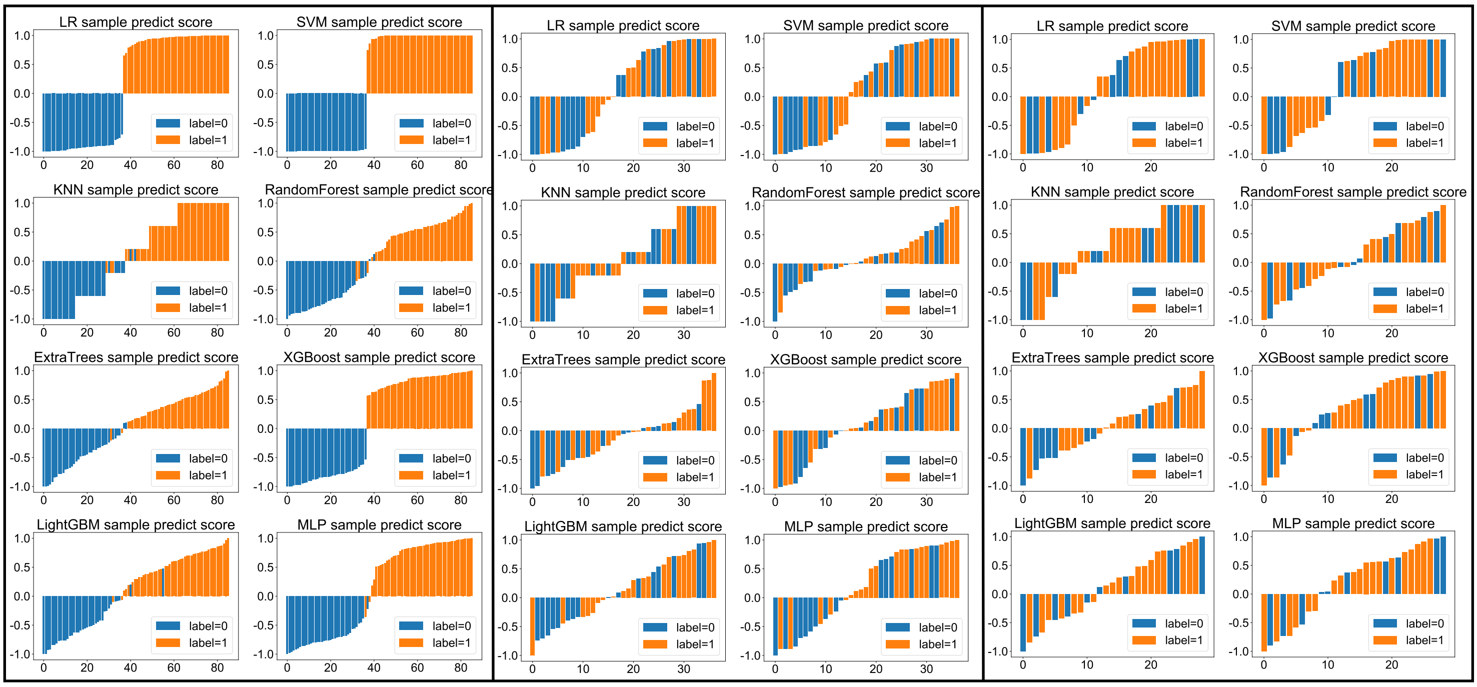

(a) (b) (c)

Supplementary Figure S22. Waterfall figure in LLLN_Fusion_Models. (a) Training cohort (TUMC). (b) Test cohort (TUMC). (c) Test cohort (GSPH). (LLLN_Fusion_Models, the models combine radiomics and deep transfer learning features based on largest short-axis lateral lymph node; RF, random forest; KNN, k-nearest neighbor; LR, logistic regression; MLP, multilayer perceptron; SVM, support vector machine; XGBoost, extreme gradient boosting; LightGBM, light gradient boosting machine; TUMC, Tianjin Union Medical Center; GSPH, Gansu Provincial Hospital.)

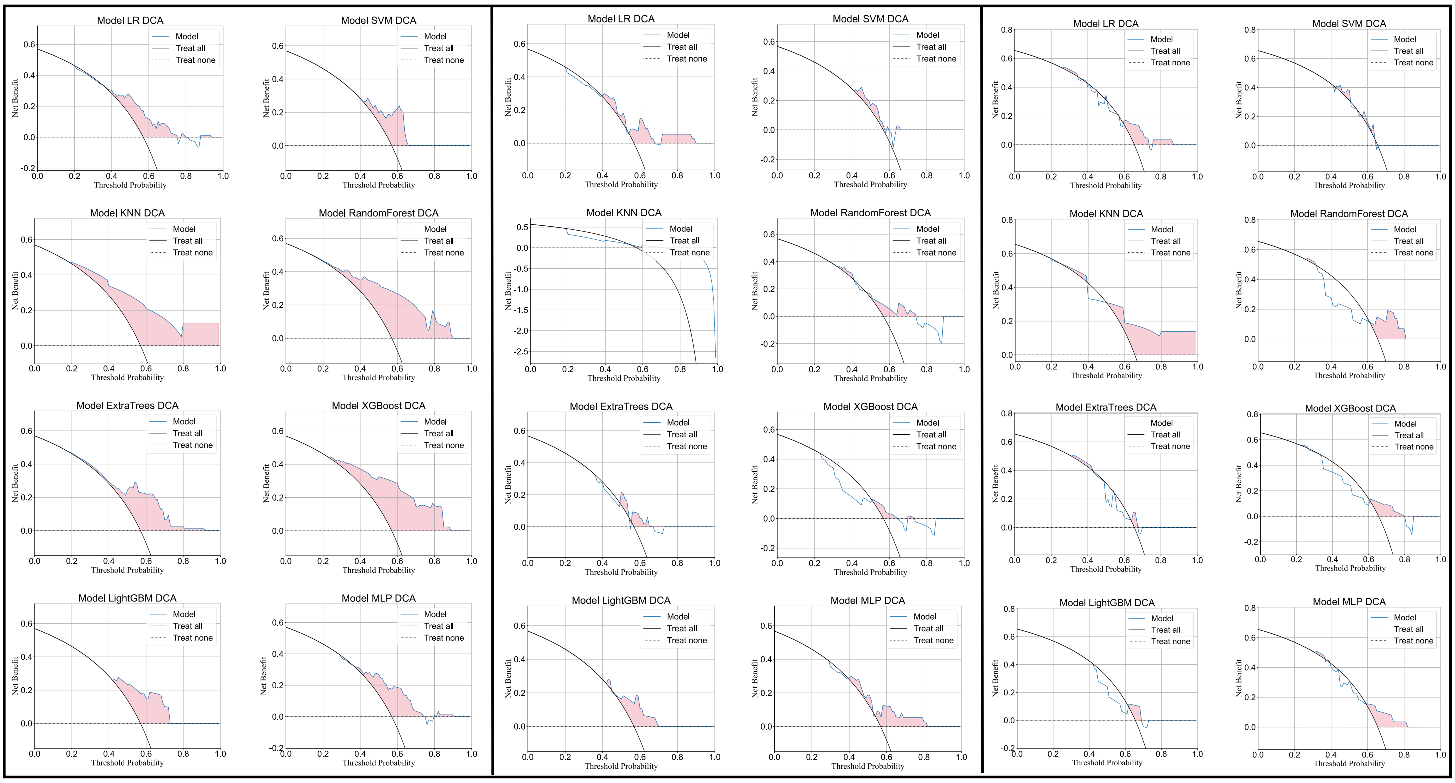

(a) (b) (c)

Supplementary Figure S23. DCA in clinical models. (a) Training cohort (TUMC). (b) Test cohort (TUMC). (c) Test cohort (GSPH). (RF, random forest; KNN, k-nearest neighbor; LR, logistic regression; MLP, multilayer perceptron; SVM, support vector machine; XGBoost, extreme gradient boosting; LightGBM, light gradient boosting machine; TUMC, Tianjin Union Medical Center; GSPH, Gansu Provincial Hospital.)

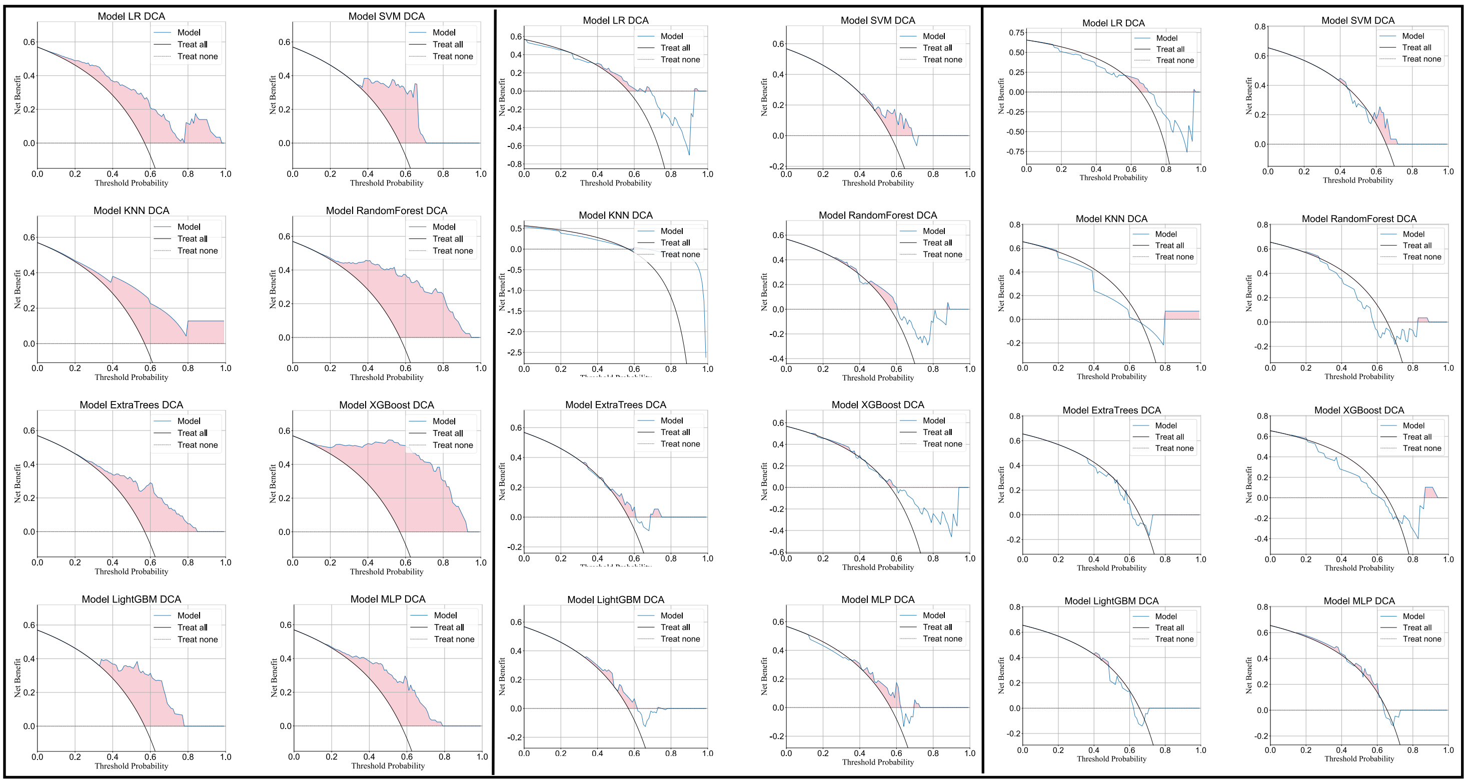

(a) (b) (c)

Supplementary Figure S24. DCA in PT_Rad_Models. (a) Training cohort (TUMC). (b) Test cohort (TUMC). (c) Test cohort (GSPH). (PT_Rad_Models, the radiomics models based on primary tumor; RF, random forest; KNN, k-nearest neighbor; LR, logistic regression; MLP, multilayer perceptron; SVM, support vector machine; XGBoost, extreme gradient boosting; LightGBM, light gradient boosting machine; TUMC, Tianjin Union Medical Center; GSPH, Gansu Provincial Hospital.)

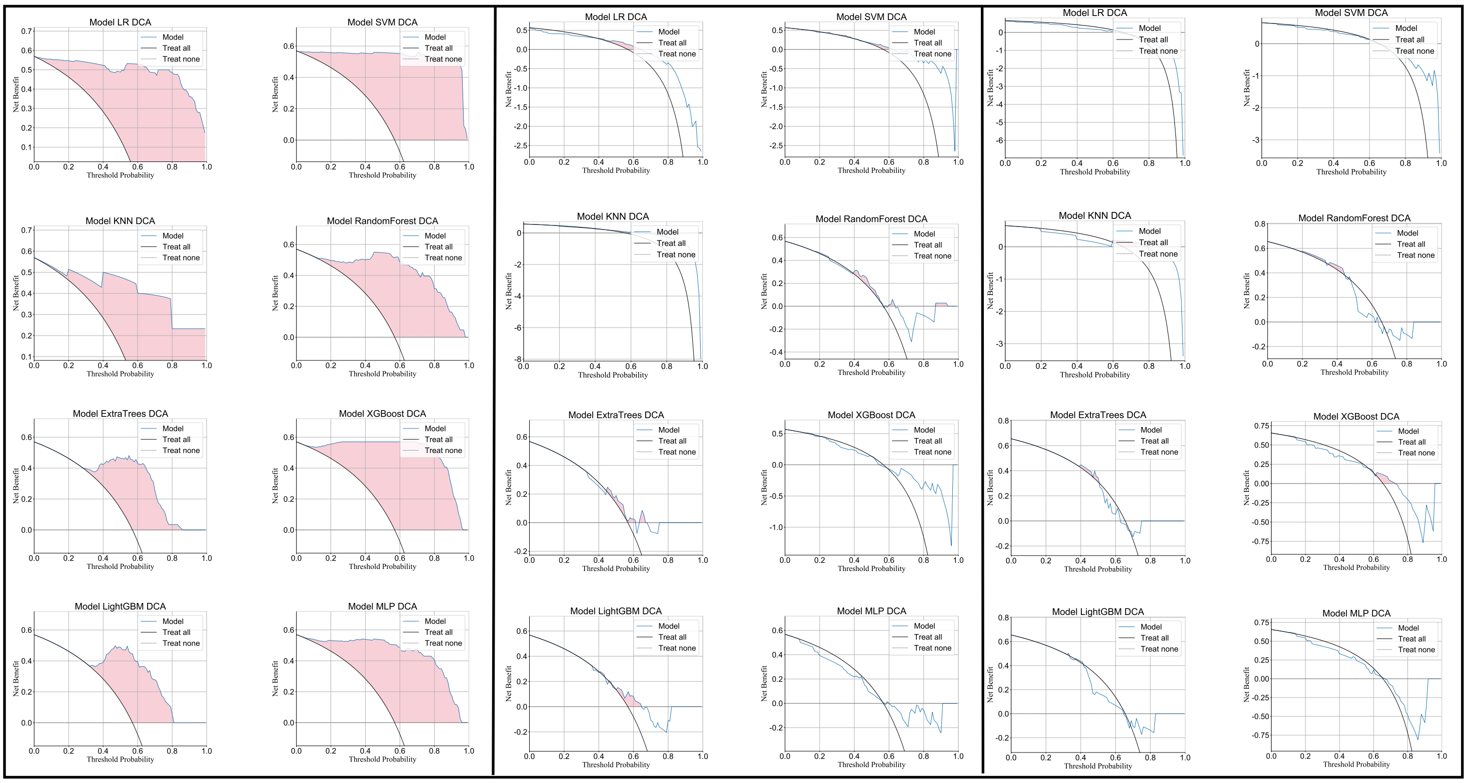

(a) (b) (c)

Supplementary Figure S25. DCA in PT_Fusion_Models. (a) Training cohort (TUMC). (b) Test cohort (TUMC). (c) Test cohort (GSPH). (PT_Fusion_Models, the models combine radiomics and deep transfer learning features based on the primary tumor; RF, random forest; KNN, k-nearest neighbor; LR, logistic regression; MLP, multilayer perceptron; SVM, support vector machine; XGBoost, extreme gradient boosting; LightGBM, light gradient boosting machine; TUMC, Tianjin Union Medical Center; GSPH, Gansu Provincial Hospital.)

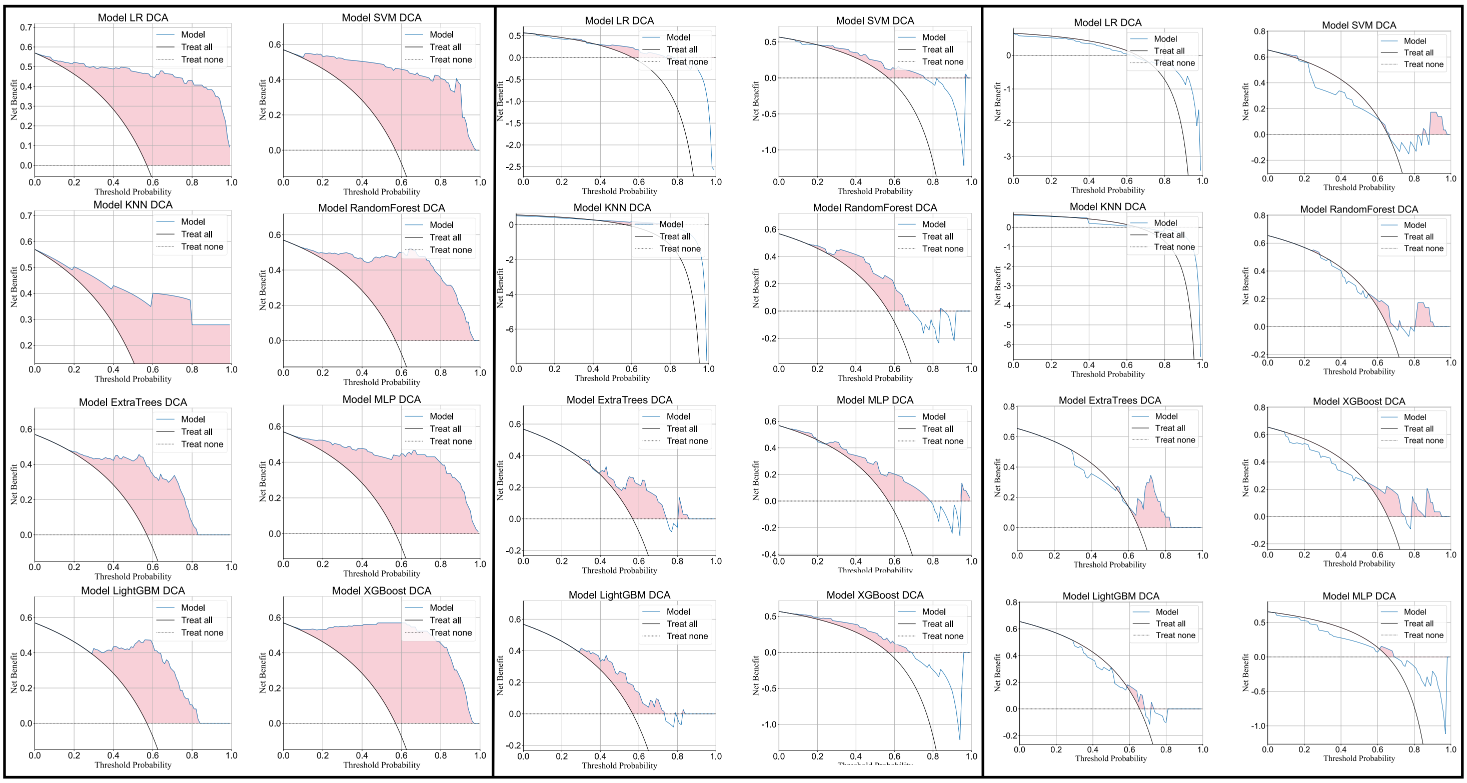

(a) (b) (c)

Supplementary Figure S26. DCA in LLLN_Rad_Models. (a) Training cohort (TUMC). (b) Test cohort (TUMC). (c) Test cohort (GSPH). (LLLN_Rad_Models, the radiomics model based on largest short-axis lateral lymph node; RF, random forest; KNN, k-nearest neighbor; LR, logistic regression; MLP, multilayer perceptron; SVM, support vector machine; XGBoost, extreme gradient boosting; LightGBM, light gradient boosting machine; TUMC, Tianjin Union Medical Center; GSPH, Gansu Provincial Hospital.)

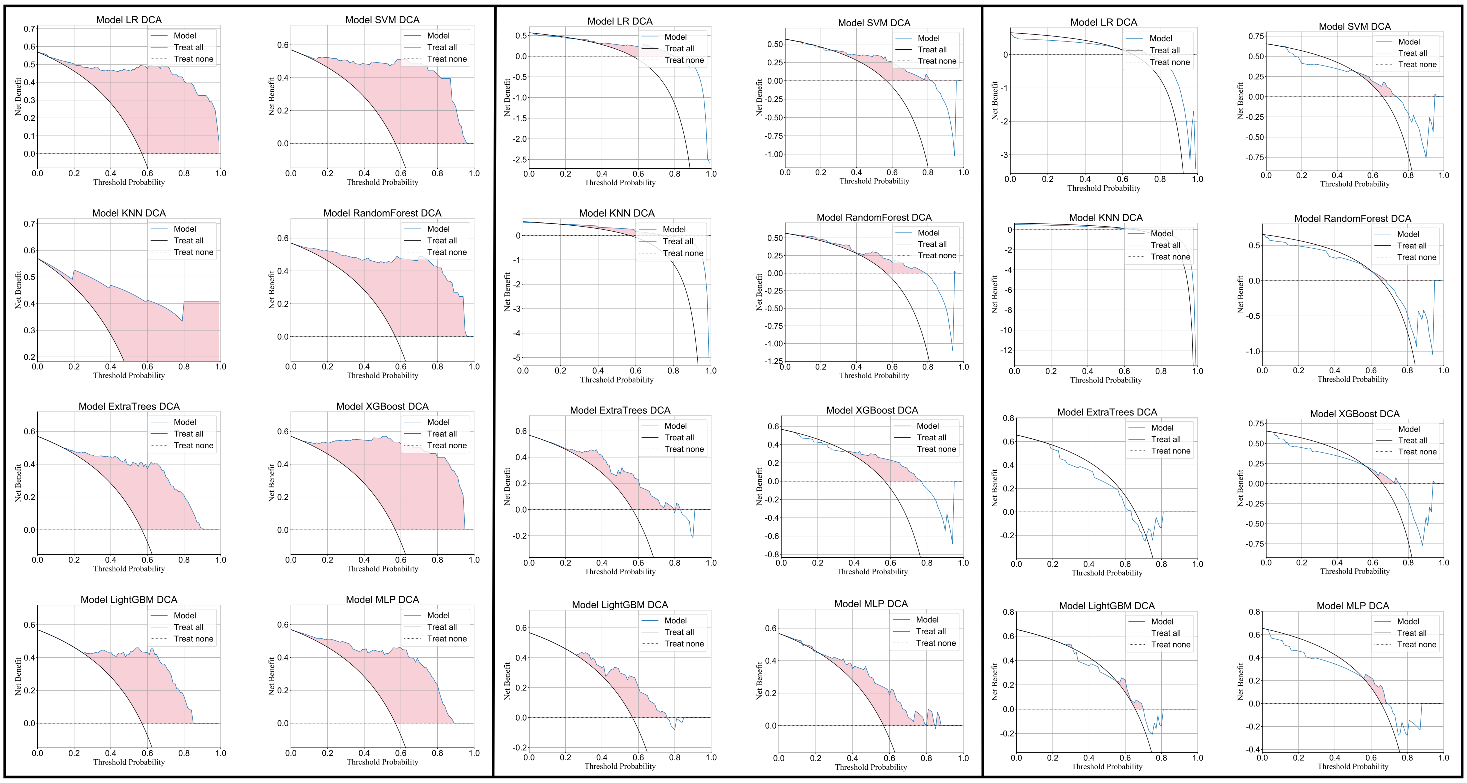

(a) (b) (c)

Supplementary Figure S27. DCA in VLLN_Rad_Models. (a) Training cohort (TUMC). (b) Test cohort (TUMC). (c) Test cohort (GSPH). (VLLN_Rad_Models, the radiomics model based on all visible lateral lymph nodes; RF, random forest; KNN, k-nearest neighbor; LR, logistic regression; MLP, multilayer perceptron; SVM, support vector machine; XGBoost, extreme gradient boosting; LightGBM, light gradient boosting machine; TUMC, Tianjin Union Medical Center; GSPH, Gansu Provincial Hospital.)

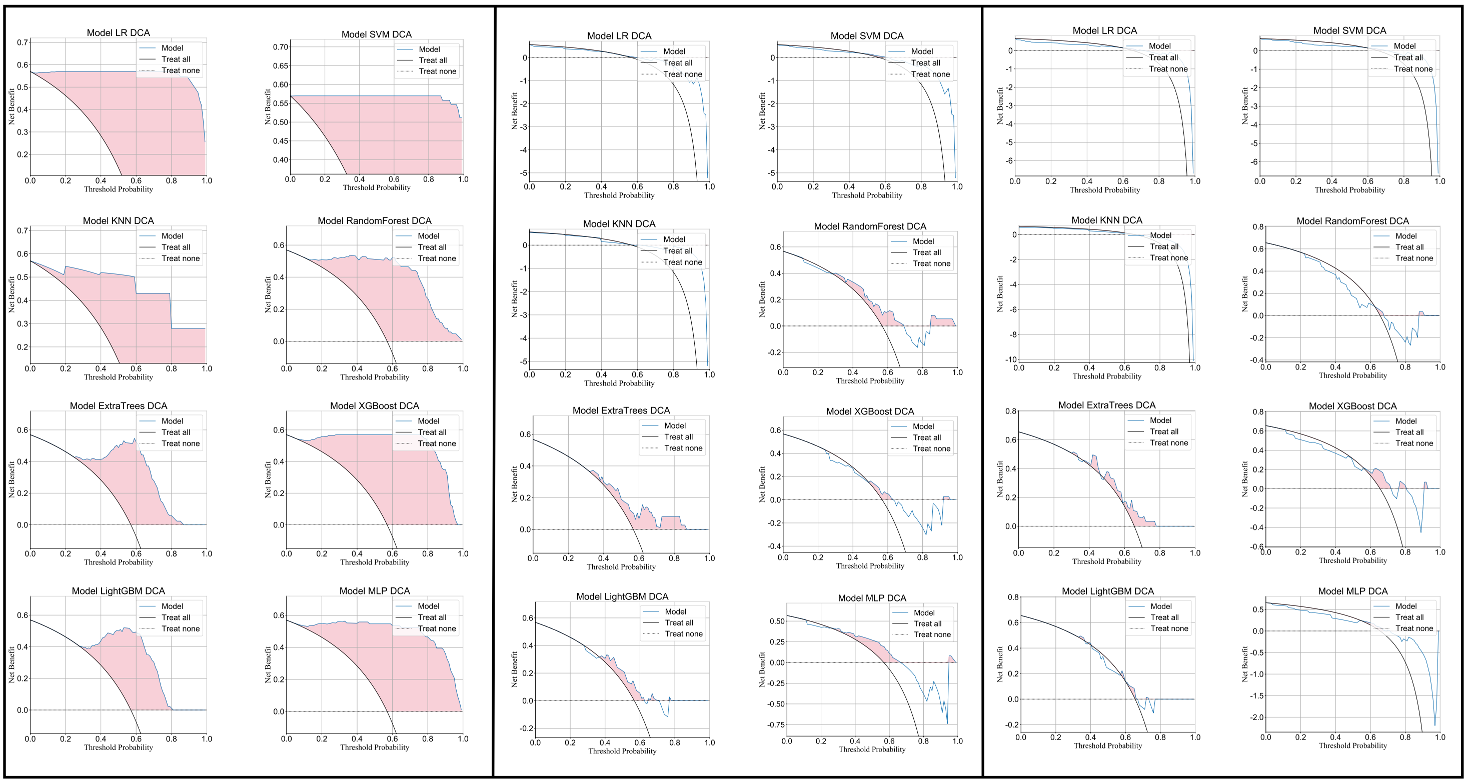

(a) (b) (c)

Supplementary Figure S28. DCA in LLLN_Fusion_Models. (a) Training cohort (TUMC). (b) Test cohort (TUMC). (c) Test cohort (GSPH). (LLLN_Fusion_Models, the models combine radiomics and deep transfer learning features based on largest short-axis lateral lymph node; RF, random forest; KNN, k-nearest neighbor; LR, logistic regression; MLP, multilayer perceptron; SVM, support vector machine; XGBoost, extreme gradient boosting; LightGBM, light gradient boosting machine; TUMC, Tianjin Union Medical Center; GSPH, Gansu Provincial Hospital.)

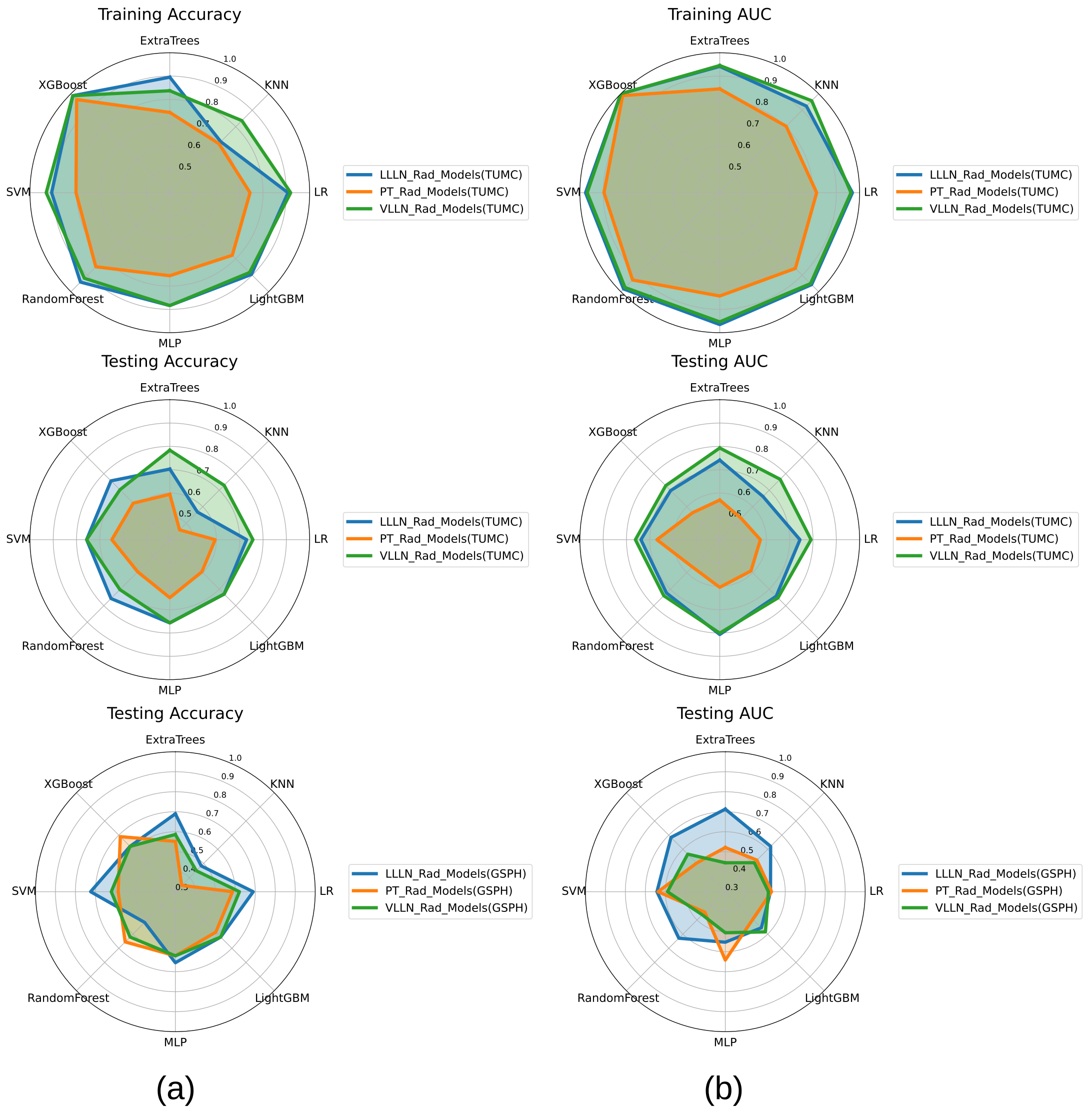

Supplementary Figure S29. Radar chart of the accuracy and AUC of the radiomics models. (a) Accuracy. (b) AUC. (AUC, area under the curve; PT_Rad_Models, radiomics models based on primary tumor; LLLN_Rad_Models, radiomics models based on largest short-axis lateral lymph node; VLLN_Rad_Models, radiomics models based on all visible lateral lymph nodes; ROC, receiver operating characteristic; RF, random forest; KNN, k-nearest neighbor; LR, logistic regression; MLP, multilayer perceptron; SVM, support vector machine; XGBoost, extreme gradient boosting; LightGBM, light gradient boosting machine; TUMC, Tianjin Union Medical Center; GSPH, Gansu Provincial Hospital.)

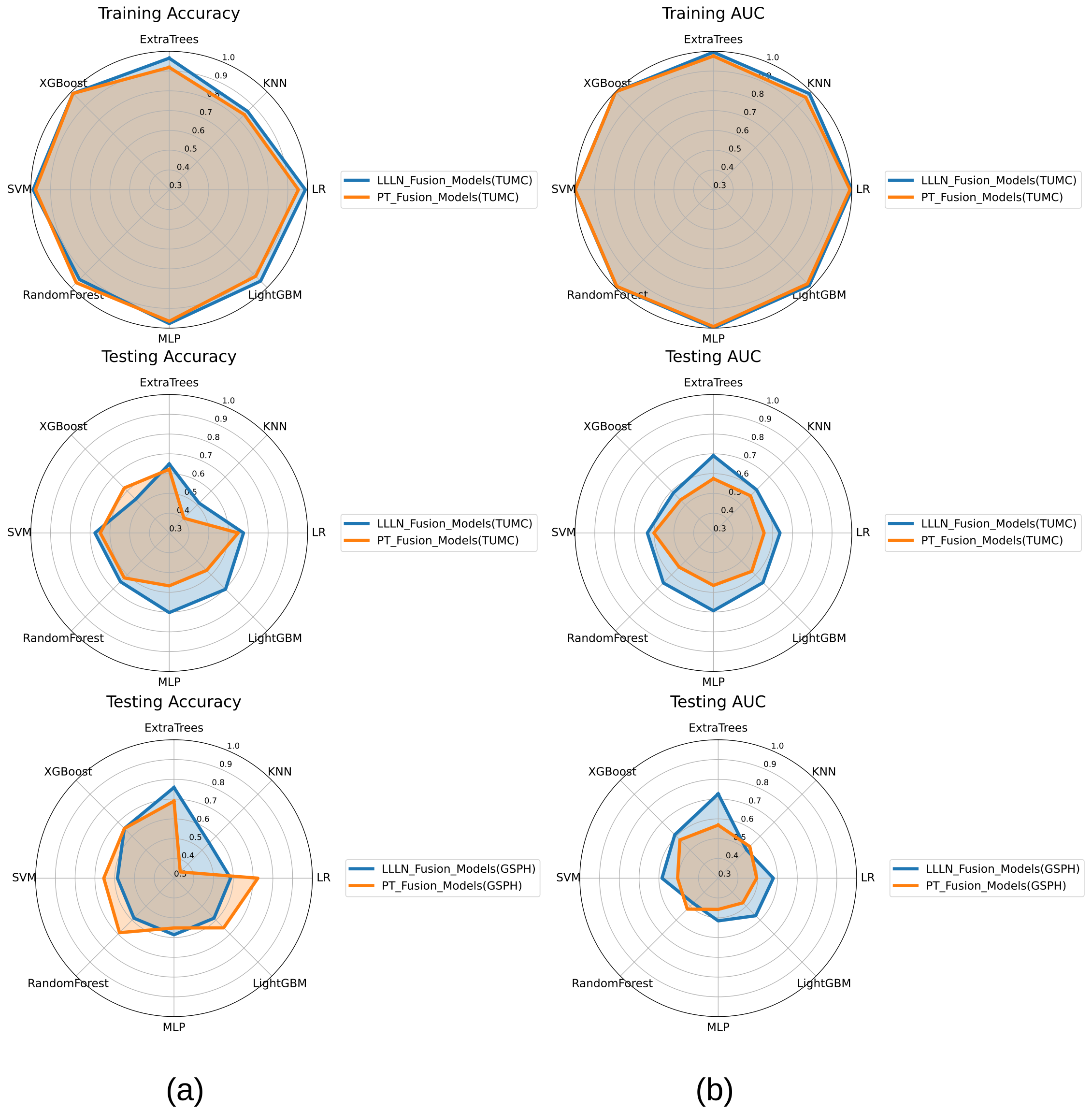

Supplementary Figure S30. Radar chart of the accuracy and AUC of the fusion models. (a) Accuracy. (b) AUC. (AUC, area under the curve; PT_Fusion_Models, the models combine radiomics and deep transfer learning features based on the primary tumor; LLLN_Fusion_Models, the models combine radiomics and deep transfer learning features based on largest short-axis lateral lymph node; RF, random forest; KNN, k-nearest neighbor; LR, logistic regression; MLP, multilayer perceptron; SVM, support vector machine; XGBoost, extreme gradient boosting; LightGBM, light gradient boosting machine; TUMC, Tianjin Union Medical Center; GSPH, Gansu Provincial Hospital.)

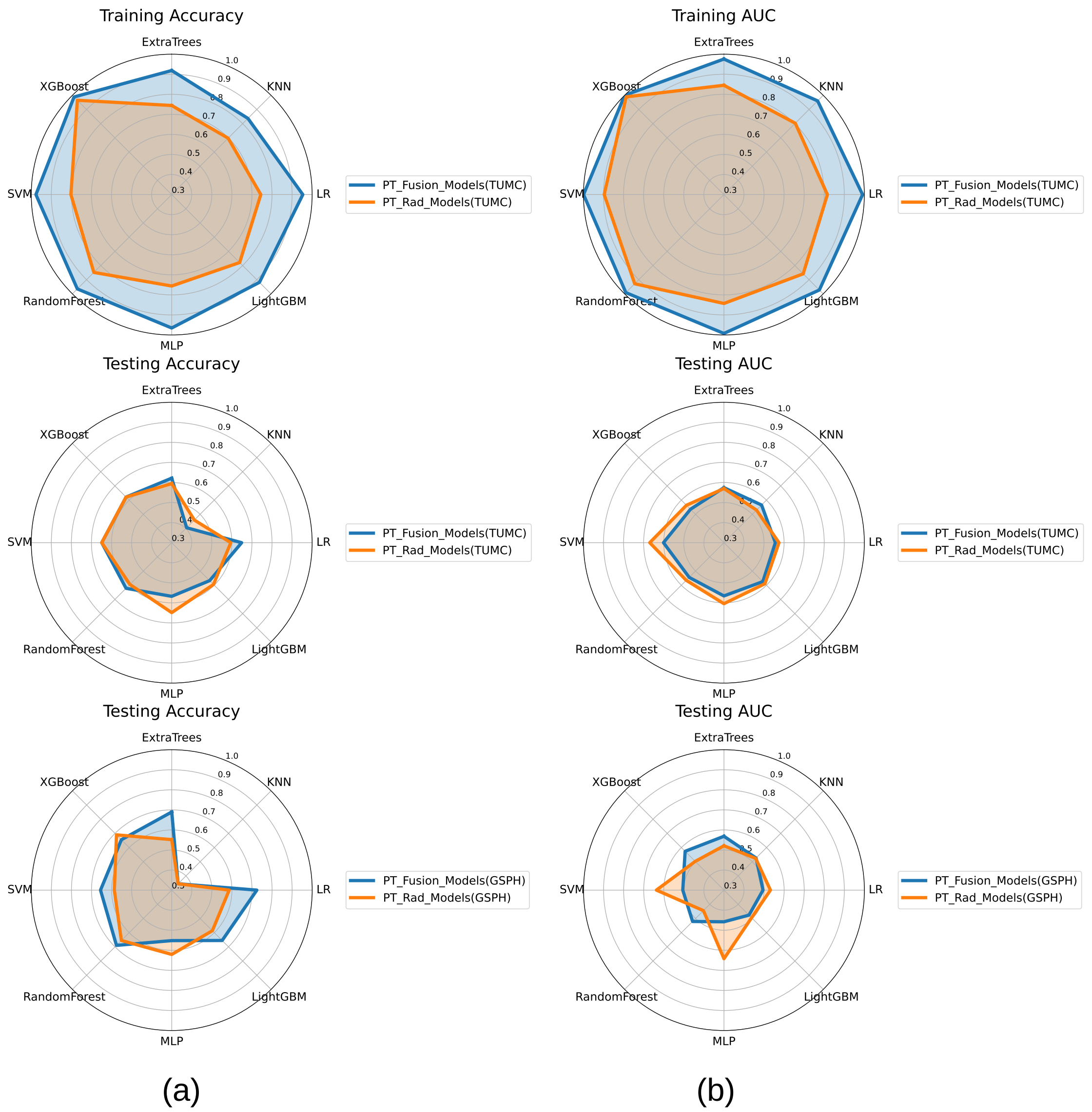

Supplementary Figure S31. Radar chart of the accuracy and AUC of the models based on primary tumor. (a) Accuracy. (b) AUC. (AUC, area under the curve; PT_Rad_Models, radiomics models based on primary tumor; PT_Fusion_Models, the models combine radiomics and deep transfer learning features based on the primary tumor; RF, random forest; KNN, k-nearest neighbor; LR, logistic regression; MLP, multilayer perceptron; SVM, support vector machine; XGBoost, extreme gradient boosting; LightGBM, light gradient boosting machine; TUMC, Tianjin Union Medical Center; GSPH, Gansu Provincial Hospital.)

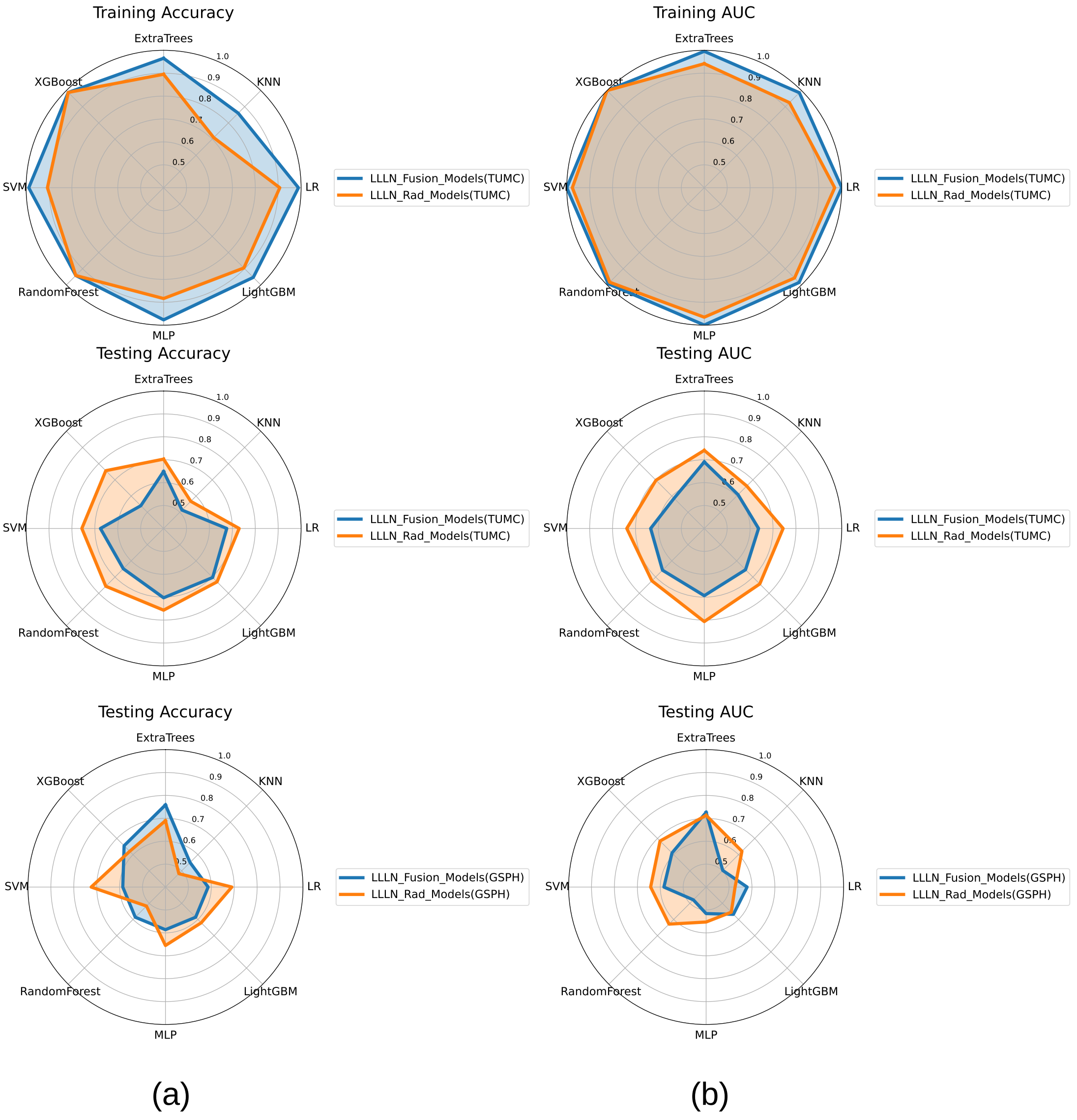

Supplementary Figure S32. Radar chart of the accuracy and AUC of the models based on largest short-axis lateral lymph node. (a) Accuracy. (b) AUC.(AUC, area under the curve; LLLN_Rad_Models, radiomics models based on largest short-axis lateral lymph node; LLLN_Fusion_Models, the models combine radiomics and deep transfer learning features based on largest short-axis lateral lymph node; RF, random forest; KNN, k-nearest neighbor; LR, logistic regression; MLP, multilayer perceptron; SVM, support vector machine; XGBoost, extreme gradient boosting; LightGBM, light gradient boosting machine; TUMC, Tianjin Union Medical Center; GSPH, Gansu Provincial Hospital.)

Supplementary Tables

Supplementary Table S1. Model performance in clinical models

Mode	Accuracy	AUC	95% CI	Sensitivity	Specificity	PPV	NPV	Precision	Recall	F1	Threshold	Task
LR	0.69	0.7	0.59 - 0.81	0.76	0.59	0.71	0.65	0.71	0.76	0.73	0.5	train(TUMC)
	0.65	0.65	0.47 - 0.84	0.76	0.5	0.67	0.62	0.67	0.76	0.71	0.46	test(TUMC)
	0.66	0.66	0.44 - 0.88	0.63	0.7	0.8	0.5	0.8	0.63	0.71	0.5	test(GSPH)
SVM	0.67	0.75	0.65 - 0.86	0.53	0.86	0.84	0.58	0.84	0.53	0.65	0.61	train(TUMC)
	0.65	0.58	0.37 - 0.78	0.86	0.37	0.64	0.67	0.64	0.86	0.73	0.45	test(TUMC)
	0.62	0.66	0.43 - 0.88	0.68	0.5	0.72	0.45	0.72	0.68	0.7	0.51	test(GSPH)
KNN	0.67	0.79	0.70 - 0.88	0.55	0.84	0.82	0.58	0.82	0.55	0.66	0.6	train(TUMC)
	0.43	0.5	0.31 - 0.68	0.05	0.94	0.5	0.43	0.5	0.05	0.09	0.8	test(TUMC)
	0.55	0.75	0.58 - 0.92	0.37	0.9	0.87	0.43	0.87	0.37	0.52	0.6	test(GSPH)
RandomForest	0.76	0.81	0.72 - 0.90	0.78	0.73	0.79	0.71	0.79	0.78	0.78	0.43	train(TUMC)
	0.68	0.68	0.50 - 0.87	0.81	0.5	0.68	0.67	0.68	0.81	0.74	0.37	test(TUMC)
	0.55	0.63	0.42 - 0.85	0.37	0.9	0.87	0.43	0.87	0.37	0.52	0.75	test(GSPH)
ExtraTrees	0.7	0.8	0.71 - 0.89	0.59	0.84	0.83	0.61	0.83	0.59	0.69	0.56	train(TUMC)
	0.62	0.57	0.38 - 0.77	0.71	0.5	0.65	0.57	0.65	0.71	0.68	0.52	test(TUMC)
	0.59	0.57	0.34 - 0.80	0.53	0.7	0.77	0.44	0.77	0.53	0.62	0.54	test(GSPH)
XGBoost	0.73	0.82	0.73 - 0.91	0.67	0.81	0.82	0.65	0.82	0.67	0.74	0.61	train(TUMC)
	0.54	0.5	0.31 - 0.69	0.48	0.62	0.62	0.48	0.62	0.48	0.54	0.58	test(TUMC)
	0.62	0.62	0.39 - 0.85	0.74	0.4	0.7	0.44	0.7	0.74	0.72	0.34	test(GSPH)
LightGBM	0.58	0.73	0.62 - 0.83	0.31	0.95	0.88	0.51	0.88	0.31	0.45	0.68	train(TUMC)
	0.57	0.68	0.50 - 0.85	0.33	0.87	0.78	0.5	0.78	0.33	0.47	0.6	test(TUMC)
	0.38	0.56	0.33 - 0.79	0.11	0.9	0.67	0.35	0.67	0.11	0.18	0.68	test(GSPH)
MLP	0.69	0.72	0.61 - 0.83	0.73	0.62	0.72	0.64	0.72	0.73	0.73	0.52	train(TUMC)
	0.65	0.65	0.47 - 0.83	0.76	0.5	0.67	0.62	0.67	0.76	0.71	0.46	test(TUMC)
	0.66	0.65	0.42 - 0.87	0.63	0.7	0.8	0.5	0.8	0.63	0.71	0.5	test(GSPH)

AUC, area under the curve; PPV, positive predictive value; NPV, negative predictive value; F1, the harmonic mean of precision and recall; RF, random forest; KNN, k-nearest neighbor; LR, logistic regression; MLP, multilayer perceptron; SVM, support vector machine; XGBoost, extreme gradient boosting; LightGBM, light gradient boosting machine; TUMC, Tianjin Union Medical Center; GSPH, Gansu Provincial Hospital.

Supplementary Table S2. Model performance of the radiomics models based on PT

Mode	Accuracy	AUC	95% CI	Sensitivity	Specificity	PPV	NPV	Precision	Recall	F1	Threshold	Task
LR	0.74	0.82	0.73 - 0.91	0.78	0.7	0.78	0.7	0.78	0.78	0.78	0.55	train (TUMC)
	0.59	0.57	0.37 - 0.78	0.52	0.69	0.69	0.52	0.69	0.52	0.59	0.69	test(TUMC)
	0.59	0.53	0.28 - 0.78	0.53	0.7	0.77	0.44	0.77	0.53	0.62	0.55	test(GSPH)
SVM	0.8	0.9	0.83 - 0.96	0.67	0.97	0.97	0.69	0.97	0.67	0.8	0.66	train(TUMC)
	0.65	0.67	0.49 - 0.85	0.52	0.81	0.79	0.57	0.79	0.52	0.63	0.63	test(TUMC)
	0.59	0.64	0.43 - 0.84	0.42	0.9	0.89	0.45	0.89	0.42	0.57	0.62	test(GSPH)
KNN	0.7	0.8	0.71 - 0.89	0.61	0.81	0.81	0.61	0.81	0.61	0.7	0.6	train(TUMC)
	0.46	0.53	0.35 - 0.71	0.1	0.94	0.67	0.44	0.67	0.1	0.17	0.8	test(TUMC)
	0.34	0.52	0.29 - 0.75	0.0	1.0	0.0	0.34	0.0	0.0	nan	1.0	test(GSPH)
RandomForest	0.85	0.93	0.88 - 0.98	0.88	0.81	0.86	0.83	0.86	0.88	0.87	0.54	train(TUMC)
	0.59	0.57	0.37 - 0.76	0.71	0.44	0.62	0.54	0.62	0.71	0.67	0.58	test(TUMC)
	0.66	0.44	0.20 - 0.69	0.95	0.1	0.67	0.5	0.67	0.95	0.78	0.27	test(GSPH)
ExtraTrees	0.74	0.85	0.76 - 0.93	0.61	0.92	0.91	0.64	0.91	0.61	0.73	0.61	train(TUMC)
	0.59	0.57	0.38 - 0.76	0.71	0.44	0.62	0.54	0.62	0.71	0.67	0.54	test(TUMC)
	0.55	0.52	0.27 - 0.77	0.47	0.7	0.75	0.41	0.75	0.47	0.58	0.58	test(GSPH)
XGBoost	0.97	0.99	0.97 - 1.0	0.98	0.95	0.96	0.97	0.96	0.98	0.97	0.53	train(TUMC)
	0.62	0.56	0.36 - 0.77	0.76	0.44	0.64	0.58	0.64	0.76	0.7	0.41	test(TUMC)
	0.69	0.5	0.25 - 0.75	0.95	0.2	0.69	0.67	0.69	0.95	0.8	0.21	test(GSPH)
LightGBM	0.78	0.86	0.78 - 0.94	0.78	0.78	0.83	0.72	0.83	0.78	0.8	0.54	train(TUMC)
	0.59	0.59	0.40 - 0.78	0.71	0.44	0.62	0.54	0.62	0.71	0.67	0.61	test(TUMC)
	0.59	0.49	0.24 - 0.75	0.63	0.5	0.71	0.42	0.71	0.63	0.67	0.54	test(GSPH)
MLP	0.76	0.84	0.76 - 0.92	0.71	0.81	0.83	0.68	0.83	0.71	0.77	0.59	train(TUMC)
	0.65	0.6	0.40 - 0.80	0.62	0.69	0.72	0.58	0.72	0.62	0.67	0.61	test(TUMC)
	0.62	0.64	0.40 - 0.88	0.58	0.7	0.79	0.47	0.79	0.58	0.67	0.57	test(GSPH)

PT, primary tumor; AUC, area under the curve; PPV, positive predictive value; NPV, negative predictive value; F1, the harmonic mean of precision and recall; RF, random forest; KNN, k-nearest neighbor; LR, logistic regression; MLP, multilayer perceptron; SVM, support vector machine; XGBoost, extreme gradient boosting; LightGBM, light gradient boosting machine; TUMC, Tianjin Union Medical Center; GSPH, Gansu Provincial Hospital.

Supplementary Table S3. Model performance of fusion (radiomics and DTL) models based on PT

Mode	Accuracy	AUC	95% CI	Sensitivity	Specificity	PPV	NPV	Precision	Recall	F1	Threshold	Task
LR	0.95	0.99	0.98 – 1.0	0.94	0.97	0.98	0.92	0.98	0.94	0.96	0.64	train(TUMC)
	0.65	0.56	0.35 - 0.76	0.76	0.5	0.67	0.62	0.67	0.76	0.71	0.23	test(TUMC)
	0.72	0.49	0.22 - 0.77	0.89	0.4	0.74	0.67	0.74	0.89	0.81	0.06	test(GSPH)
SVM	0.98	1.0	1.0 – 1.0	0.96	1.0	1.0	0.95	1.0	0.96	0.98	0.72	train(TUMC)
	0.65	0.6	0.4 - 0.8	0.86	0.37	0.64	0.67	0.64	0.86	0.73	0.36	test(TUMC)
	0.66	0.51	0.23 - 0.78	0.79	0.4	0.71	0.5	0.71	0.79	0.75	0.37	test(GSPH)
KNN	0.84	0.96	0.93 - 0.99	0.73	0.97	0.97	0.73	0.97	0.73	0.84	0.6	train(TUMC)
	0.41	0.57	0.37 - 0.76	0.1	0.81	0.4	0.41	0.4	0.1	0.15	0.8	test(TUMC)
	0.34	0.53	0.31 - 0.74	0.05	0.9	0.5	0.33	0.5	0.05	0.1	0.8	test(GSPH)
RandomForest	0.97	0.99	0.98 – 1.0	0.98	0.95	0.96	0.97	0.96	0.98	0.97	0.54	train(TUMC)
	0.62	0.54	0.34 - 0.75	0.86	0.31	0.62	0.62	0.62	0.86	0.72	0.48	test(TUMC)
	0.69	0.52	0.26 - 0.78	0.89	0.3	0.71	0.6	0.71	0.89	0.79	0.43	test(GSPH)
ExtraTrees	0.92	0.98	0.95 – 1.0	0.92	0.92	0.94	0.89	0.94	0.92	0.93	0.55	train(TUMC)
	0.62	0.57	0.38 - 0.77	0.71	0.5	0.65	0.57	0.65	0.71	0.68	0.51	test(TUMC)
	0.69	0.57	0.32 - 0.82	0.89	0.3	0.71	0.6	0.71	0.89	0.79	0.48	test(GSPH)
XGBoost	0.99	1.0	1.0 – 1.0	0.98	1.0	1.0	0.97	1.0	0.98	0.99	0.7	train(TUMC)
	0.62	0.54	0.34 - 0.73	0.9	0.25	0.61	0.67	0.61	0.9	0.73	0.23	test(TUMC)
	0.66	0.57	0.32 - 0.82	0.74	0.5	0.74	0.5	0.74	0.74	0.74	0.34	test(GSPH)
LightGBM	0.92	0.97	0.94 – 1.0	0.9	0.95	0.96	0.87	0.96	0.9	0.93	0.54	train(TUMC)
	0.57	0.57	0.38 - 0.77	0.57	0.56	0.63	0.5	0.63	0.57	0.6	0.54	test(TUMC)
	0.66	0.48	0.23 - 0.72	0.89	0.2	0.68	0.5	0.68	0.89	0.77	0.42	test(GSPH)
MLP	0.97	0.99	0.98 – 1.0	0.98	0.95	0.96	0.97	0.96	0.98	0.97	0.51	train(TUMC)
	0.57	0.57	0.37 - 0.76	0.38	0.81	0.73	0.5	0.73	0.38	0.5	0.77	test(TUMC)
	0.55	0.46	0.2 - 0.72	0.53	0.6	0.71	0.4	0.71	0.53	0.61	0.6	test(GSPH)

PT, primary tumor; DTL, deep transfer learning; AUC, area under the curve; PPV, positive predictive value; NPV, negative predictive value; F1, the harmonic mean of precision and recall; RF, random forest; KNN, k-nearest neighbor; LR, logistic regression; MLP, multilayer perceptron; SVM, support vector machine; XGBoost, extreme gradient boosting; LightGBM, light gradient boosting machine; TUMC, Tianjin Union Medical Center; GSPH, Gansu Provincial Hospital.

Supplementary Table S4. Model performance of the radiomics models based on LLLN

Mode	Accuracy	AUC	95% CI	Sensitivity	Specificity	PPV	NPV	Precision	Recall	F1	Threshold	Task
LR	0.91	0.97	0.94 - 1.0	0.86	0.97	0.98	0.84	0.98	0.86	0.91	0.65	train(TUMC)
	0.73	0.74	0.58 - 0.91	0.81	0.62	0.74	0.71	0.74	0.81	0.77	0.31	test(TUMC)
	0.69	0.53	0.27 - 0.78	0.84	0.4	0.73	0.57	0.73	0.84	0.78	0.28	test(GSPH)
SVM	0.91	0.98	0.95 - 1.0	0.92	0.89	0.92	0.89	0.92	0.92	0.92	0.52	train(TUMC)
	0.76	0.74	0.56 - 0.91	0.86	0.62	0.75	0.77	0.75	0.86	0.8	0.29	test(TUMC)
	0.72	0.64	0.42 - 0.86	0.95	0.3	0.72	0.75	0.72	0.95	0.82	0.17	test(GSPH)
KNN	0.71	0.93	0.88 - 0.98	0.49	1.0	1.0	0.6	1.0	0.49	0.66	0.8	train(TUMC)
	0.57	0.66	0.48 - 0.84	0.38	0.81	0.73	0.5	0.73	0.38	0.5	0.8	test(TUMC)
	0.48	0.62	0.38 - 0.86	0.47	0.5	0.64	0.33	0.64	0.47	0.55	0.4	test(GSPH)
RandomForest	0.94	0.98	0.96 - 1.0	0.9	1.0	1.0	0.88	1.0	0.9	0.95	0.65	train(TUMC)
	0.76	0.72	0.54 - 0.90	0.9	0.56	0.73	0.82	0.73	0.9	0.81	0.44	test(TUMC)
	0.52	0.63	0.42 - 0.84	0.26	1.0	1.0	0.42	1.0	0.26	0.42	0.81	test(GSPH)
ExtraTrees	0.9	0.94	0.90 - 0.99	0.92	0.86	0.9	0.89	0.9	0.92	0.91	0.53	train(TUMC)
	0.7	0.74	0.58 - 0.91	0.62	0.81	0.81	0.62	0.81	0.62	0.7	0.63	test(TUMC)
	0.69	0.71	0.52 - 0.90	0.58	0.9	0.92	0.53	0.92	0.58	0.71	0.7	test(GSPH)
XGBoost	0.99	1.0	1.0 - 1.0	0.98	1.0	1.0	0.97	1.0	0.98	0.99	0.63	train(TUMC)
	0.76	0.7	0.50 - 0.89	0.9	0.56	0.73	0.82	0.73	0.9	0.81	0.27	test(TUMC)
	0.62	0.68	0.48 - 0.89	0.58	0.7	0.79	0.47	0.79	0.58	0.67	0.72	test(GSPH)
LightGBM	0.9	0.96	0.92 - 1.0	0.84	0.97	0.98	0.82	0.98	0.84	0.9	0.59	train(TUMC)
	0.73	0.74	0.57 - 0.91	0.86	0.56	0.72	0.75	0.72	0.86	0.78	0.44	test(TUMC)
	0.62	0.56	0.32 - 0.79	0.74	0.4	0.7	0.44	0.7	0.74	0.72	0.5	test(GSPH)
MLP	0.88	0.97	0.93 - 1.0	0.8	1.0	1.0	0.79	1.0	0.8	0.89	0.67	train(TUMC)
	0.76	0.81	0.66 - 0.95	0.81	0.69	0.77	0.73	0.77	0.81	0.79	0.33	test(TUMC)
	0.66	0.55	0.31 - 0.79	0.84	0.3	0.7	0.5	0.7	0.84	0.76	0.22	test(GSPH)

LLLN, largest short-axis lateral lymph node; AUC, area under the curve; PPV, positive predictive value; NPV, negative predictive value; F1, the harmonic mean of precision and recall; RF, random forest; KNN, k-nearest neighbor; LR, logistic regression; MLP, multilayer perceptron; SVM, support vector machine; XGBoost, extreme gradient boosting; LightGBM, light gradient boosting machine; TUMC, Tianjin Union Medical Center; GSPH, Gansu Provincial Hospital.

Supplementary Table S5. Model performance of the radiomics models based on VLLN

Mode	Accuracy	AUC	95% CI	Sensitivity	Specificity	PPV	NPV	Precision	Recall	F1	Threshold	Task
LR	0.92	0.96	0.93 - 1.0	0.86	1.0	1.0	0.84	1.0	0.86	0.92	0.68	train(TUMC)
	0.76	0.79	0.63 - 0.95	0.71	0.81	0.83	0.68	0.83	0.71	0.77	0.64	test(TUMC)
	0.62	0.52	0.26 - 0.77	0.63	0.6	0.75	0.46	0.75	0.63	0.69	0.71	test(GSPH)
SVM	0.93	0.97	0.93 - 1.0	0.88	1.0	1.0	0.86	1.0	0.88	0.93	0.64	train(TUMC)
	0.76	0.76	0.58 - 0.94	0.71	0.81	0.83	0.68	0.83	0.71	0.77	0.56	test(TUMC)
	0.62	0.59	0.36 - 0.82	0.63	0.6	0.75	0.46	0.75	0.63	0.69	0.67	test(GSPH)
KNN	0.84	0.96	0.92 - 0.99	0.71	1.0	1.0	0.73	1.0	0.71	0.83	0.8	train(TUMC)
	0.73	0.77	0.60 - 0.93	0.71	0.75	0.79	0.67	0.79	0.71	0.75	0.4	test(TUMC)
	0.45	0.51	0.28 - 0.73	0.37	0.6	0.64	0.33	0.64	0.37	0.47	0.8	test(GSPH)
RandomForest	0.92	0.97	0.95 - 1.0	0.9	0.95	0.96	0.87	0.96	0.9	0.93	0.59	train(TUMC)
	0.7	0.74	0.57 - 0.91	0.71	0.69	0.75	0.65	0.75	0.71	0.73	0.52	test(TUMC)
	0.62	0.46	0.22 - 0.71	0.79	0.3	0.68	0.43	0.68	0.79	0.73	0.35	test(GSPH)
ExtraTrees	0.84	0.95	0.90 - 0.99	0.73	0.97	0.97	0.73	0.97	0.73	0.84	0.63	train(TUMC)
	0.78	0.79	0.63 - 0.95	0.95	0.56	0.74	0.9	0.74	0.95	0.83	0.38	test(TUMC)
	0.59	0.44	0.20 - 0.69	0.74	0.3	0.67	0.37	0.67	0.74	0.7	0.43	test(GSPH)
XGBoost	0.99	1.0	1.0 - 1.0	0.98	1.0	1.0	0.97	1.0	0.98	0.99	0.52	train(TUMC)
	0.7	0.73	0.55 - 0.90	0.62	0.81	0.81	0.62	0.81	0.62	0.7	0.52	test(TUMC)
	0.62	0.57	0.34 - 0.79	0.63	0.6	0.75	0.46	0.75	0.63	0.69	0.74	test(GSPH)
LightGBM	0.88	0.95	0.91 - 0.99	0.84	0.95	0.95	0.81	0.95	0.84	0.89	0.56	train(TUMC)
	0.73	0.75	0.59 - 0.92	0.71	0.75	0.79	0.67	0.79	0.71	0.75	0.49	test(TUMC)
	0.62	0.58	0.33 - 0.84	0.63	0.6	0.75	0.46	0.75	0.63	0.69	0.6	test(GSPH)
MLP	0.88	0.95	0.92 - 0.99	0.82	0.97	0.98	0.8	0.98	0.82	0.89	0.61	train(TUMC)
	0.76	0.8	0.65 - 0.95	0.76	0.75	0.8	0.71	0.8	0.76	0.78	0.48	test(TUMC)
	0.62	0.51	0.25 - 0.76	0.63	0.6	0.75	0.46	0.75	0.63	0.69	0.65	test(GSPH)

VLLN, visible lateral lymph nodes; AUC, area under the curve; PPV, positive predictive value; NPV, negative predictive value; F1, the harmonic mean of precision and recall; RF, random forest; KNN, k-nearest neighbor; LR, logistic regression; MLP, multilayer perceptron; SVM, support vector machine; XGBoost, extreme gradient boosting; LightGBM, light gradient boosting machine; TUMC, Tianjin Union Medical Center; GSPH, Gansu Provincial Hospital.

Supplementary Table S6. Model performance of fusion (radiomics and DTL) models based on LLLN

Mode	Accuracy	AUC	95% CI	Sensitivity	Specificity	PPV	NPV	Precision	Recall	F1	Threshold	Task
LR	0.99	1.0	1.0 - 1.0	0.98	1.0	1.0	0.97	1.0	0.98	0.99	0.83	train(TUMC)
	0.68	0.64	0.45 - 0.82	0.81	0.5	0.68	0.67	0.68	0.81	0.74	0.18	test(TUMC)
	0.59	0.58	0.34 - 0.82	0.47	0.8	0.82	0.44	0.82	0.47	0.6	0.89	test(GSPH)
SVM	0.99	1.0	1.0 - 1.0	0.98	1.0	1.0	0.97	1.0	0.98	0.99	0.87	train(TUMC)
	0.68	0.63	0.44 - 0.82	0.86	0.44	0.67	0.7	0.67	0.86	0.75	0.08	test(TUMC)
	0.59	0.58	0.34 - 0.83	0.53	0.7	0.77	0.44	0.77	0.53	0.62	0.86	test(GSPH)
KNN	0.86	0.99	0.97 - 1.0	0.76	1.0	1.0	0.76	1.0	0.76	0.86	0.6	train(TUMC)
	0.51	0.61	0.42 - 0.80	0.52	0.5	0.58	0.44	0.58	0.52	0.55	0.4	test(TUMC)
	0.55	0.5	0.26 - 0.74	0.68	0.3	0.65	0.33	0.65	0.68	0.67	0.4	test(GSPH)
RandomForest	0.94	0.99	0.98 - 1.0	0.9	1.0	1.0	0.88	1.0	0.9	0.95	0.61	train(TUMC)
	0.65	0.66	0.47 - 0.84	0.81	0.44	0.65	0.64	0.65	0.81	0.72	0.47	test(TUMC)
	0.59	0.48	0.25 - 0.71	0.74	0.3	0.67	0.37	0.67	0.74	0.7	0.43	test(GSPH)
ExtraTrees	0.97	1.0	0.99 - 1.0	0.94	1.0	1.0	0.92	1.0	0.94	0.97	0.59	train(TUMC)
	0.65	0.69	0.52 - 0.86	0.76	0.5	0.67	0.62	0.67	0.76	0.71	0.48	test(TUMC)
	0.76	0.73	0.52 - 0.94	0.89	0.5	0.77	0.71	0.77	0.89	0.83	0.45	test(GSPH)
XGBoost	0.99	1.0	1.0 - 1.0	0.98	1.0	1.0	0.97	1.0	0.98	0.99	0.77	train(TUMC)
	0.54	0.59	0.40 - 0.78	0.24	0.94	0.83	0.48	0.83	0.24	0.37	0.86	test(TUMC)
	0.66	0.61	0.38 - 0.84	0.68	0.6	0.76	0.5	0.76	0.68	0.72	0.63	test(GSPH)
LightGBM	0.95	0.99	0.96 - 1.0	0.96	0.95	0.96	0.95	0.96	0.96	0.96	0.53	train(TUMC)
	0.7	0.65	0.47 - 0.84	0.86	0.5	0.69	0.73	0.69	0.86	0.77	0.45	test(TUMC)
	0.59	0.57	0.32 - 0.81	0.58	0.6	0.73	0.43	0.73	0.58	0.65	0.58	test(GSPH)
MLP	0.98	1.0	1.0 - 1.0	0.96	1.0	1.0	0.95	1.0	0.96	0.98	0.46	train(TUMC)
	0.7	0.69	0.51 - 0.87	0.76	0.62	0.73	0.67	0.73	0.76	0.74	0.51	test(TUMC)
	0.59	0.52	0.27 - 0.76	0.63	0.5	0.71	0.42	0.71	0.63	0.67	0.62	test(GSPH)

LLLN, largest short-axis lateral lymph node; DTL, deep transfer learning; AUC, area under the curve; PPV, positive predictive value; NPV, negative predictive value; F1, the harmonic mean of precision and recall; RF, random forest; KNN, k-nearest neighbor; LR, logistic regression; MLP, multilayer perceptron; SVM, support vector machine; XGBoost, extreme gradient boosting; LightGBM, light gradient boosting machine; TUMC, Tianjin Union Medical Center; GSPH, Gansu Provincial Hospital.

Configuration file for Pyradiomics

```
imageType:
  Original: {}
  LoG:
    # If you include sigma values >5, remember to also increase the padDistance. Because of
resampling to (2, 2, 2), the
    # use of sigmas < 2 mm is not recommended.
    sigma: [2.0, 3.0, 4.0, 5.0]
  Wavelet: {}

featureClass:
  # redundant Compactness 1, Compactness 2 an Spherical Disproportion features are
disabled by default, they can be
  # enabled by specifying individual feature names (as is done for glcm) and including them
in the list.
  shape:
  firstorder:
  glcm:  # Disable SumAverage by specifying all other GLCM features available
    - 'Autocorrelation'
    - 'JointAverage'
    - 'ClusterProminence'
    - 'ClusterShade'
    - 'ClusterTendency'
    - 'Contrast'
    - 'Correlation'
    - 'DifferenceAverage'
    - 'DifferenceEntropy'
    - 'DifferenceVariance'
    - 'JointEnergy'
    - 'JointEntropy'
    - 'Imc1'
    - 'Imc2'
    - 'Idm'
    - 'Idmn'
    - 'Id'
    - 'Idn'
    - 'InverseVariance'
    - 'MaximumProbability'
    - 'SumEntropy'
    - 'SumSquares'
  glrlm:
  glszm:
  gldm:
```

  ngtdm:

setting:
  # Normalization:
  # MR signal is usually relative, with large differences between scanners and vendors. By normalizing the image before
  # feature calculation, this confounding effect may be reduced. However, if only one specific scanner is used, or the
  # images reflect some absolute world value (e.g. ADC maps, T2maps (NOT T2 weighted)), consider disabling the
  # normalization.
  normalize: true
  normalizeScale: 100 # This allows you to use more or less the same bin width.

  # Resampling:
  # If slices are very thin (~1mm), such as in 3D scanned (isotropic) volumes, resampledPixelSpacing may be reduced to
  # (1, 1, 1). Furthermore, in case of isotropic volumes, consider disabling resampling.
  # On a side note: increasing the resampled spacing forces PyRadiomics to look at more coarse textures, which may or
  # may not increase accuracy and stability of your extracted features.
  interpolator: 'sitkBSpline'
  resampledPixelSpacing: [2, 2, 2]

  # Mask validation:
  # correctMask and geometryTolerance are not needed, as both image and mask are resampled, if you expect very small
  # masks, consider to enable a size constraint by uncommenting settings below:
  #minimumROIDimensions: 2
  #minimumROISize: 50

  # Image discretization:
  # The ideal number of bins is somewhere in the order of 16-128 bins. A possible way to define a good binwidt is to
  # extract firstorder:Range from the dataset to analyze, and choose a binwidth so, that range/binwidth remains approximately
  # in this range of bins.
  binWidth: 5

  # first order specific settings:
  # When normalizing, gray values below the mean will be negative. Shifting by 300 (3 StdDevs * 100) ensures that the
  # majority of voxels is positive (only outliers >3 SD lower than the mean will be negative).
  voxelArrayShift: 300

  # Misc:
  # default label value. Labels can also be defined in the call to featureextractor.execute, as a commandline argument,
  # or in a column "Label" in the input csv (batchprocessing)
  label: 1
